# Combination of Whole Genome Sequencing, Linkage, and Functional Studies Implicates a Missense Mutation in Titin as a Cause of Autosomal Dominant Cardiomyopathy With Features of Left Ventricular Noncompaction

**DOI:** 10.1161/CIRCGENETICS.116.001431

**Published:** 2016-10-18

**Authors:** Robert Hastings, Carin P. de Villiers, Charlotte Hooper, Liz Ormondroyd, Alistair Pagnamenta, Stefano Lise, Silvia Salatino, Samantha J.L. Knight, Jenny C. Taylor, Kate L. Thomson, Linda Arnold, Spyros D. Chatziefthimiou, Petr V. Konarev, Matthias Wilmanns, Elisabeth Ehler, Andrea Ghisleni, Mathias Gautel, Edward Blair, Hugh Watkins, Katja Gehmlich

**Keywords:** cardiomyopathy, left ventricular noncompaction, missense mutation, telethonin, titin, whole genome sequencing

## Abstract

**Background—:**

High throughput next-generation sequencing techniques have made whole genome sequencing accessible in clinical practice; however, the abundance of variation in the human genomes makes the identification of a disease-causing mutation on a background of benign rare variants challenging.

**Methods and Results—:**

Here we combine whole genome sequencing with linkage analysis in a 3-generation family affected by cardiomyopathy with features of autosomal dominant left ventricular noncompaction cardiomyopathy. A missense mutation in the giant protein titin is the only plausible disease-causing variant that segregates with disease among the 7 surviving affected individuals, with interrogation of the entire genome excluding other potential causes. This A178D missense mutation, affecting a conserved residue in the second immunoglobulin-like domain of titin, was introduced in a bacterially expressed recombinant protein fragment and biophysically characterized in comparison to its wild-type counterpart. Multiple experiments, including size exclusion chromatography, small-angle x ray scattering, and circular dichroism spectroscopy suggest partial unfolding and domain destabilization in the presence of the mutation. Moreover, binding experiments in mammalian cells show that the mutation markedly impairs binding to the titin ligand telethonin.

**Conclusions—:**

Here we present genetic and functional evidence implicating the novel A178D missense mutation in titin as the cause of a highly penetrant familial cardiomyopathy with features of left ventricular noncompaction. This expands the spectrum of titin’s roles in cardiomyopathies. It furthermore highlights that rare titin missense variants, currently often ignored or left uninterpreted, should be considered to be relevant for cardiomyopathies and can be identified by the approach presented here.

## Abstract 2

Supplemental Digital Content is available in the text.

Cardiomyopathies are a diverse group of diseases affecting the heart muscle^1^; many of them are inherited and transmitted in autosomal dominant patterns. The first cardiomyopathy genes were identified by genome-wide linkage analysis in large families.^2^ In practice, however, the small size of most families, or even the availability of members of larger families, often limits the power of linkage analysis. Recently, high throughput next-generation sequencing techniques have become widely accessible, making whole genome sequencing (WGS) cost-effective and time-effective. However, the abundance of variation in the human genome^3^ makes it difficult to distinguish rare benign variants from rare disease-causing mutations in an isolated individual, even with growing knowledge of variants in population cohorts (eg, >60 000 sequenced exomes in the Exome Aggregation Consortium (ExAC) database, http://exac.broadinstitute.org/). Next-generation sequencing poses, therefore, a significant clinical challenge: the capability to assess variants as pathogenic lags significantly behind variant identification, especially for nonsynonymous point mutations.^4,5^ Algorithmic predictors are currently unable to accurately assess their exact impact on protein–protein interactions or even on protein folding. Experimental validation of genetic variants is, therefore, an increasingly indispensable component of next-generation sequencing discoveries.

**Editorial, see p 392**

**Clinical Perspective on p 435**

In the current study, we combine WGS with linkage analysis in a medium-sized family affected by cardiomyopathy, with features of left ventricular noncompaction cardiomyopathy (LVNC). By performing WGS in 2 family members, filtering against variants seen in normal population cohorts and using linkage information derived from single nucleotide polymorphism (SNP) arrays of 13 family members, we could identify a missense variant in the titin gene (*TTN*) as the most plausible cause of disease in the family. Functional data generated from biophysical and protein-binding experiments on this titin missense variant provide further support of a causative role in cardiomyopathy through domain misfolding and destabilization, resulting in impaired binding to the ligand telethonin (also known as t-cap).

## Methods

### Clinical Evaluation

The study was approved by the Oxfordshire Research Ethics Committee B (REC Ref 09/H0605/3), and all subjects gave informed consent. A 3-generational family with history of cardiomyopathy was recruited. Clinical assessment and genetic studies were performed in available family members, who had clinical examination, ECG, echocardiography (with contrast agent where appropriate), and cardiac magnetic resonance imaging, if possible. Diagnosis of cardiomyopathy was based on established criteria. The diagnosis of LVNC was based on published criteria from echocardiographic or cardiac magnetic resonance imaging^6,7^: the compaction ratio, that is, the ratio of the thickness of noncompacted to compacted myocardium >2.3 measured on magnetic resonance imaging in diastole or >2.0 on echocardiography in systole, was used to diagnose LVNC.

### Genetic Studies

SNP array genotyping was performed using the Illumina HumanCytoSNP-12v1 BeadChip (Illumina, San Diego, CA), containing nearly 300 000 genetic markers, according to the manufacturer’s protocols. A refined subset of roughly 24 000 SNPs in approximate linkage equilibrium was generated using the software PLINK v1.07^8^ and the HapMap genotype file available from the PLINK website (http://pngu.mgh.harvard.edu/purcell/plink/). Linkage analysis of the SNP subset was performed using MERLIN v1.1.2,^9^ specifying an autosomal dominant disease model. Genomic intervals with logarithm of the odds scores >0, compatible with segregation of variants in these regions, were selected for downstream analyses.

WGS was performed on genomic DNA extracted from peripheral blood as part of the WGS500 project as described previously.^10^

Sequence reads from the affected individuals were mapped to the human reference genome (hs37d5 version of build 37) using STAMPY.^11^ Duplicate reads were removed with PICARD (http://broadinstitute.github.io/picard/). The software Platypus (version 0.8.1, default parameters)^12^ was used jointly on the two.bam files to call SNPs and short (<50 bp) indels across both samples.

All the 5 946 161 identified variants were annotated with an in-house pipeline based on the Variant Effect Predictor Ensembl framework (version 77).^13^ Several additional databases were used to integrate the information provided by Variant Effect Predictor (Table I in the Data Supplement). Known associations with diseases were screened using HGMD (http://www.hgmd.cf.ac.uk/ac/index.php) and ClinVar.^14^

Variants were filtered by in-house Python scripts based on criteria outlined in Table I in the Data Supplement (steps 1–10), followed by manual inspection (steps 11–13). The variants remaining after step 10 are documented in Results and in Tables II and III in the Data Supplement. Confirmatory Sanger sequencing was performed with the primers listed in Table IV in the Data Supplement.

Both SNP and WGS data were interrogated also for clinically relevant copy number variants using Nexus Copy Number 7.5.2 Discovery Edition (BioDiscovery, Hawthorne, CA; see Methods in the Data Supplement).

### Functional Characterization of the Titin Missense Variant

The mutation was introduced into human titin Z1Z2 constructs (amino acids 1–196, accession no ACN81321.1) for bacterial and mammalian expression using Quikchange II XL (Agilent) with primers given in Table IV in the Data Supplement. Bacterial expression and purification was performed as previously described.^15^ Size exclusion chromatography–Tridetector analysis (light scattering, refractive index, and UV absorbance), small-angle x ray scattering experiments, circular dichroism spectroscopy, and thermolysin digests were essentially performed as described,^15–18^ and experimental details are given in the Data Supplement.

Neonatal rat cardiomyocyte (NRC) cultures were established and transfected^16^ using hemagglutinin-tagged expression constructs and counterstained for titin T12 epitope^19^ or telethonin (mouse monoclonal antibody, Santa Cruz) 48 hours post transfection and analyzed by confocal microscopy.

Glutathione S-transferase (GST) pulldown assays were performed as described^20^ using mammalian expression constructs for telethonin amino acids 1 to 90 and 1 to 167 fused to GST and titin Z1Z2 fused to GFP (pEGFP-N1, Clontech) in transfected COS-1 cells. Förster Resonance Energy Transfer experiments from transfected COS-1 cells and the assessment of reduced protein stability in NRC and COS-1 cells are described in the Data Supplement.

## Results

**Figure 1.**
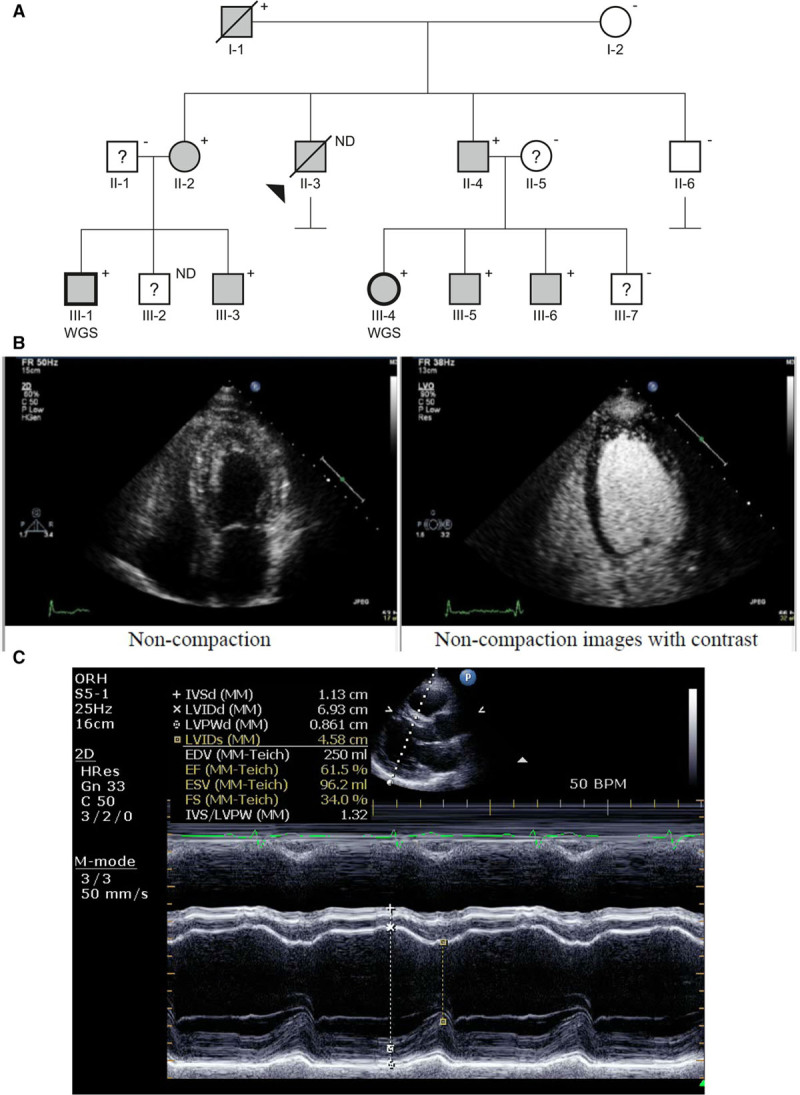
**A**, Pedigree of the family; males depicted as squares; females, circles; slanted symbols, deceased individuals. Clinically affected individuals are marked in gray, unaffected are shown in white, and ? means unclassified clinical status. The presence of the *TTN* p.A178D mutation is indicated (+ indicates present; −, absent; ND, not determined.) Individuals selected for whole genome sequencing (WGS) are marked with thicker symbols (III-1 and III-4). **B**, Echocardiogram images showing the characteristic spongy appearance of noncompaction in individual II-2 with and without contrast. **C**, Echocardiogram image from individual II-4 showing significant dilatation, but maintaining a thickened myocardium and preserved ejection fraction.

The proband was a 20-year-old male (II-3 in Figure 1A) who died suddenly in hospital in 1970 having presented with rapidly decompensating congestive heart failure; at postmortem, his heart (680 g) had evidence of dilatation and both macroscopic and microscopic hypertrophy but no myocyte disarray. His brother (II-4) was later found to have an enlarged heart with wall thickness at the upper limit of normal and marked hypertrabeculation. The proband’s sister (II-2) presented with a non–ST-segment–elevation myocardial infarct because of coronary embolus at the age of 61 years. LVNC with mild left ventricular dilatation and apical hypertrophy was diagnosed at this time (Figure 1B and 1C). Cascade screening identified the same condition in further family members with consistent clinical features of adult onset cardiomyopathy with features of LVNC. Five affected family members had sufficient noncompaction to meet the diagnostic criteria for LVNC, while 3 others with early or mild disease had lesser extent of hypertrabeculation but clear evidence of cardiomyopathy with left ventricular dilatation or systolic dysfunction (Figure 1A and Table 1; Figure I in the Data Supplement). Aside from the proband who had advanced congestive failure, there were no arrhythmic features in any affected family member, nor were there any extracardiac (eg, neuromuscular) manifestations.

**Table 1.**
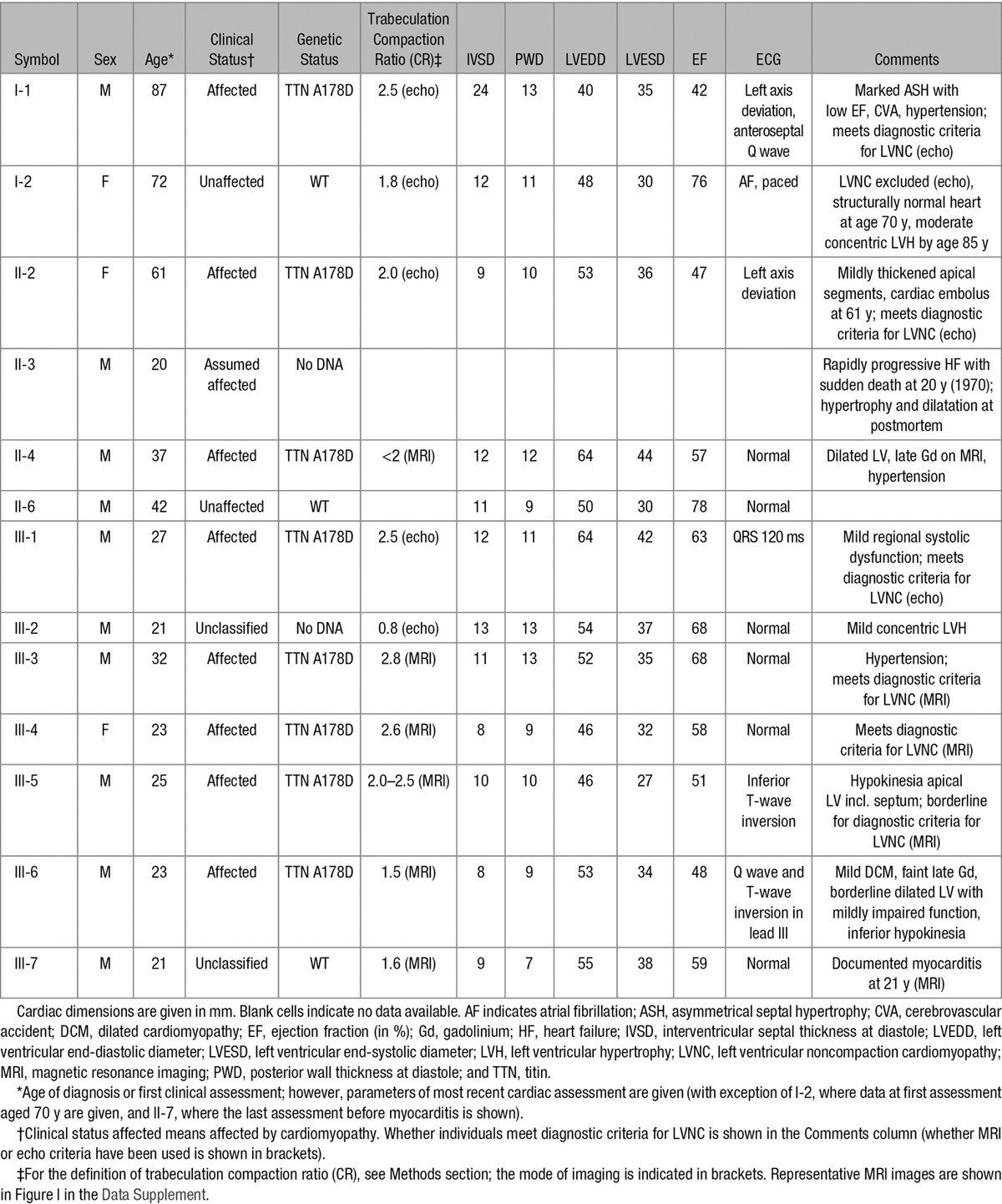
Summary of Clinical Findings

### Identification of TTN Mutation A178D Segregating With Disease

Affected first cousins III-1 and III-4 were selected for WGS. Sequencing was performed by Illumina Cambridge as 100-bp paired-end reads to a mean coverage of 56.9× and 52.0×, respectively, such that 99% of the genome was covered at 20× or more in both samples, identifying 5 946 161 variants shared by the 2 individuals. In addition, SNP arrays were performed on all individuals of the family (except II-3 and III-2; Figure 1A). Neither the SNP array nor WGS data revealed likely causative copy number variants.

Genomic regions identical by descent were identified through linkage analysis (see Methods and Figure II in the Data Supplement), and out of the 100 789 candidate variants within the 3 linkage regions (on chromosomes 2, 9, and 16), potentially pathogenic ones were selected based on an autosomal dominant model, caused by a rare heterozygous mutation. Variants were filtered accordingly by in-house Python scripts, and the remaining 6 variants were manually inspected (Table II in the Data Supplement). Four of them were excluded: one is assumed to be an artifact because of an incorrect transcript being present in Ensembl and another variant did not segregate with disease in the family; 2 splice variants were predicted to be silent (at positions -5 and -3 of a 3′ splice junction, respectively; for details, see Table III in the Data Supplement). Only 2 final candidate variants were considered conceivably linked to the phenotype: missense changes in *PDP2* and *TTN*, respectively (Table II in the Data Supplement). *PDP2* codes for pyruvate dehyrogenase phosphatase catalytic subunit 2 and has low expression levels in the heart. Although the change E316K is predicted to be damaging by Polyphen and SIFT algorithms (Table II in the Data Supplement), a heterozygous loss-of-function in this enzyme would not be expected to produce a phenotype, and indeed, heterozygous loss-of-function mutations in *PDP1* are clinically silent.^21^ The variant is not plausible as a cause of a penetrant-dominant disorder because it is found 6× in 121 412 alleles in the ExAC database. Six instances would equal at least 10% of all expected LVNC cases in ExAC, assuming a maximal prevalence of 1:1000 for the disease.^22^ This seems to be an implausibly high percentage for a novel, unpublished disease-causing variant. In support, in the 2 largest clinical cardiomyopathy cohorts published to date, the most common reported pathogenic variant (*MYBPC3*, p.Arg502Trp) detected in 104 out of 6179 hypertrophic cardiomyopathy cases (1.7%, 95% confidence interval 1.4%–2.0%) was only observed 3× in ExAC (3/120 674), with all other pathogenic variants for hypertrophic cardiomyopathy or dilated cardiomyopathy (DCM) being present 0 or 1 time only.^23^

The second variant is found in *TTN*, the gene that codes for titin, an abundant skeletal muscle and heart-specific protein with crucial functions^24,25^ (and reviewed in Gerull^26^ et al). Mutations in titin have been associated with cardiomyopathy and skeletal myopathy (reviewed in Chauveau et al^27^). The identified missense variant c.533C>A in *TTN*, which codes for a p.A178D change at the amino acid level, is absent in ExAC. Sanger sequencing confirmed the cosegregation of the heterozygous mutation with disease in all affected individuals of the family (Figure 1; Figure IIIA in the Data Supplement; logarithm of the odds score 2.1). Thus, comprehensive whole genome analysis reveals this as the most plausible causative mutation in the family.

### Functional Studies

#### Prediction of Deleterious Effects of the Mutation

**Figure 2.**
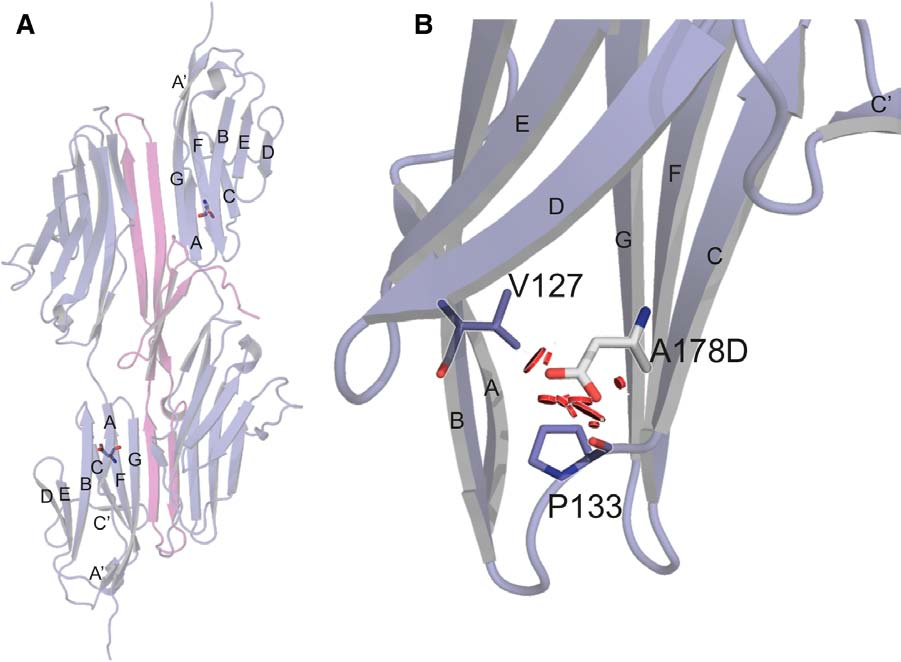
**A**, Position of the *TTN* p. A178D on a structural model (pdb: 1YA5) of the titin Z1Z2 domains (purple) in complex with telethonin (pink). **B**, Close-up of the site of mutation. The red discs show van der Waals overlaps or steric clashing that A178D is predicted to cause with valine127 and proline133. The figures of the crystal structure were generated by Pymol (http://www.pymol.org).

Each single molecule of the giant protein titin spans half a sarcomere from the Z disk to the M band.^28^ The first 2 immunoglobulin-like domains (Z1Z2) of titin are located in the Z disk and form a superstable complex with telethonin.^29^ The A178 position is evolutionarily well conserved back to zebrafish and lamprey. Additionally, A178 is located in a highly conserved structural section (Figure IIIB in the Data Supplement), the β-strand F of the second immunoglobulin domain of Z1Z2, neighboring the β-strand G of titin Z2, which forms a strong and extended interaction with the β-strands of telethonin^30^ (Figure 2A). The A178D mutation is predicted to directly affect the β-strands B and C and the loop connecting the β-strands B and C because of steric hindrance of D178 with V127 and P133, respectively (Figure 2B). Thus, the insertion of a charged residue in this position is likely to have significant impact on the secondary structure of this domain and could potentially cause misfolding of the protein.

#### Altered Protein Characteristics of Purified Titin Z1Z2 A178D Recombinant Fragment

**Figure 3.**
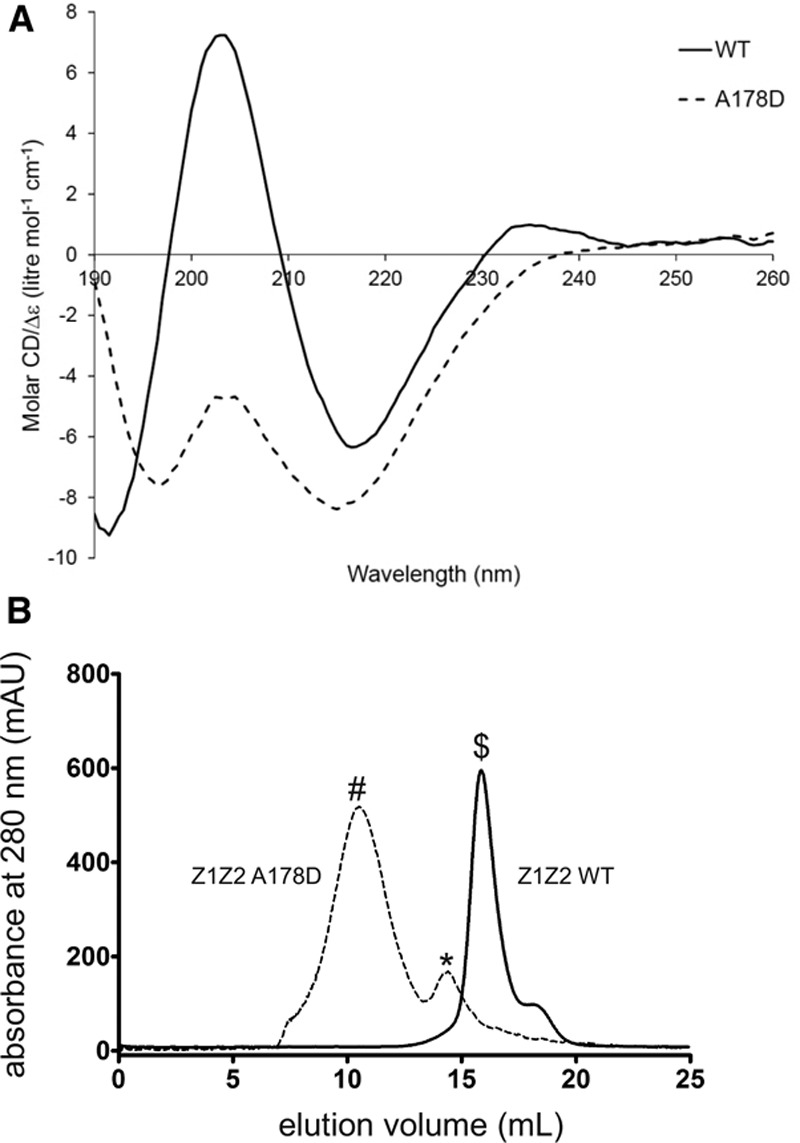
**A**, Circular dichroism (CD) spectroscopy of purified titin Z1Z2 fragments (WT solid line and A178D dashed line). **B**, Size exclusion chromatography for titin Z1Z2 fragments (WT solid line and A178D dashed line). Z1Z2 WT elutes as monomeric protein ($), whereas peaks corresponding to dimer (*) and higher molecular aggregates (#) are observed for Z1Z2 A178D.

To assess how the A178D mutation affects the folding and stability of the protein, recombinant titin Z1Z2 WT and A178D were expressed in *Escherichia* *coli* and purified under native conditions. Of note, the yield of the soluble protein fraction was consistently lower for A178D compared with WT preparations, despite equal total expression levels (data not shown). Circular dichroism spectroscopy demonstrated a typical β-sheet signature for WT Z1Z2 (Figure 3A). In contrast, the spectrum for Z1Z2 A178D differs significantly: although the characteristic negative band at 216 nm is still present, but slightly shifted, there was no significant positive band at around 200 nm. The absence of this band, associated with β-sheet conformation, and the presence of a negative peak at around 198 nm, characteristic of random coil structures, indicate that the Z1Z2 A178D mutant is partially unfolded.

In support, thermal denaturation experiments for Z1Z2 A178D showed high fluorescence signal already at low temperatures, suggesting solvent exposed hydrophobic residues because of partial unfolding. No melting temperature can be deducted for titin Z1Z2 A178D, in contrast to the WT protein, which has a melting temperature of 62°C, typical for immunoglobulin domains (Figure IV in the Data Supplement). Small-angle x ray scattering experiments confirmed the presence of unfolded parts/flexible domains in Z1Z2 A178D, as shown by the Kratky plot (Figure VA in the Data Supplement), whereas Z1Z2 WT displays a typical profile for folded structures.

The domain destabilization as a consequence of partial unfolding is evidenced by the formation of higher oligomers (≈20-mers) for the Z1Z2 A178D mutant in vitro. Size exclusion chromatography and Tridetector analysis revealed that in contrast to the monomeric Z1Z2 WT, the A178D mutant eluted in 2 peaks, corresponding predominantly to higher molecular aggregates and to a lesser extent to dimeric protein (Figure 3B and Table 2). Small-angle x ray scattering measurements also confirmed that Z1Z2 WT is monomeric, whereas Z1Z2 A178D is found in a higher oligomeric state (Table 2; Figure VB in the Data Supplement).

**Table 2.**
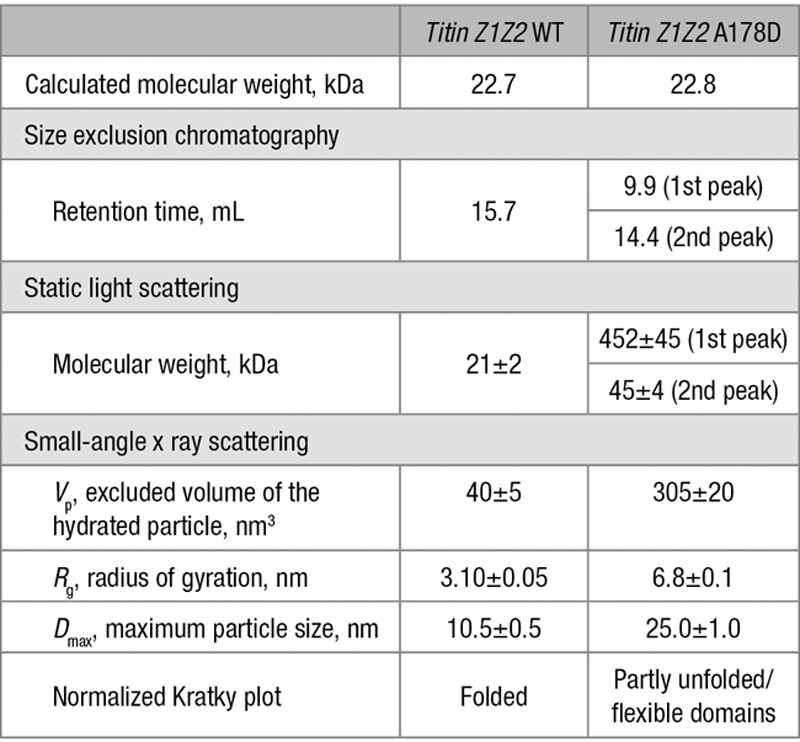
Biophysical Characterization of Recombinant Z1Z2 WT and A178D Protein Fragments

In conclusion, the mutation A178D leads to partial misfolding of bacterially expressed Z1Z2 protein fragment.

#### Reduced Stability of Titin Z1Z2 A178D as a Consequence of the Partial Misfolding

**Figure 4.**
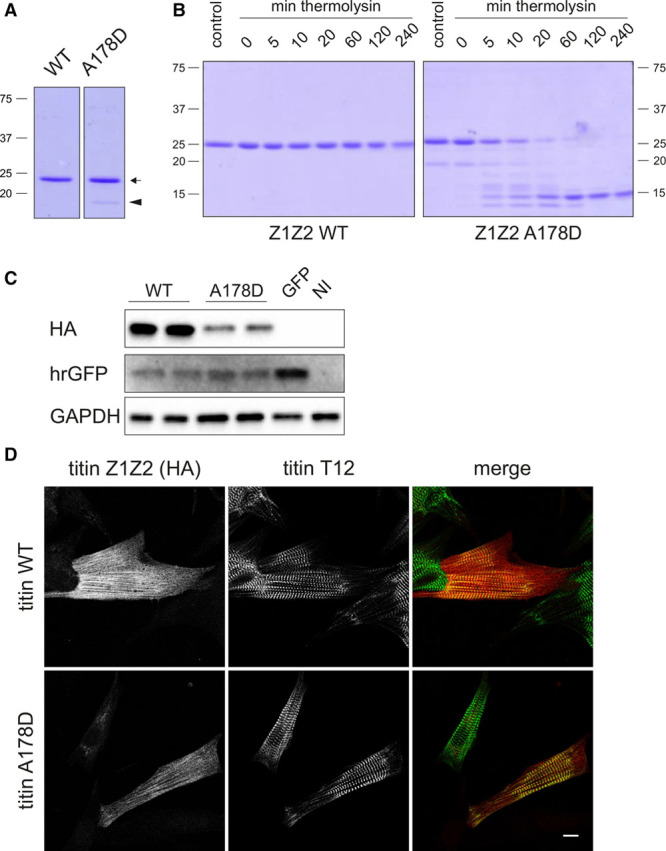
Destabilization of the titin Z1Z2 fragment in the presence of the A178D mutation. **A**, Denaturing gel-electrophoresis of purified titin Z1Z2 fragments (WT and A178D) expressed in *Escherichia* *coli.* The WT fragment is detected as a single band of 23 kDa (arrow). Only for Z1Z2 A178D, a degradation product (arrowhead) is observed. The position of marker proteins and their size (in kD) is indicated. **B**, Titin Z1Z2 protein fragments (**left**, WT; **right**, A178D) were incubated with protease thermolysin for the length indicated. Control: titin Z1Z2 protein sample without thermolysin. A stable degradation fragment of ≈15 kDa is observed for the mutant titin Z1Z2. The position of marker proteins and their size (in kD) is indicated. **C**, Decreased stability of titin Z1Z2 A178D in neonatal rat cardiomyocytes (NRC). NRC were infected in duplicates with adenoviral particles for hemagglutinin (HA)-tagged titin Z1Z2 (WT or A178D, MOI 5). Infection with parental empty vector (GFP) and noninfected cells (NI) served as controls. Steady-state titin fragment protein amount was assayed by Western blotting for the HA tag. Probing for humanized Renilla reniformis green fluorescent protein (hrGFP) served as infection control, and probing for endogenous GAPDH served as loading control. Despite equal infection rates (for confirmatory control experiments see Figure VIA in the Data Supplement), less titin A178D protein fragment was detected, indicating reduced stability in NRC. **D**, Localization of titin Z1Z2 in NRC. Cells were transfected with constructs coding for HA-tagged titin Z1Z2 WT (top, first row) or titin Z1Z2 A178D (bottom, first row) mutant protein fragment and counterstained for endogenous titin with T12 antibody (middle row, Z-disk proximal epitope, but not recognizing the transfected titin Z1Z2 protein fragment). Merged images are shown in the third row; HA, in red; endogenous titin, in green. Scale bar represents 10 μm.

When performing denaturing gel electrophoresis, a degradation product was observed exclusively for Z1Z2 A178D preparations (arrowhead in Figure 4A), and on thermolysin treatment, only Z1Z2 A178D showed rapid degradation, whereas Z1Z2 WT was resistant to the protease treatment (Figure 4B). In addition, Z1Z2 A178D showed reduced stability when expressed in neonatal rat cardiomyocytes and COS-1 cells (Figure 4C; Figure VI in the Data Supplement), suggesting that the mutation destabilizes Z1Z2 also in a physiological, cellular environment. However, formation of large aggregates was not observed in transfected cells expressing Z1Z2 A178D (Figure 4D; Figure VII in the Data Supplement).

#### Impaired Binding to Telethonin

**Figure 5.**
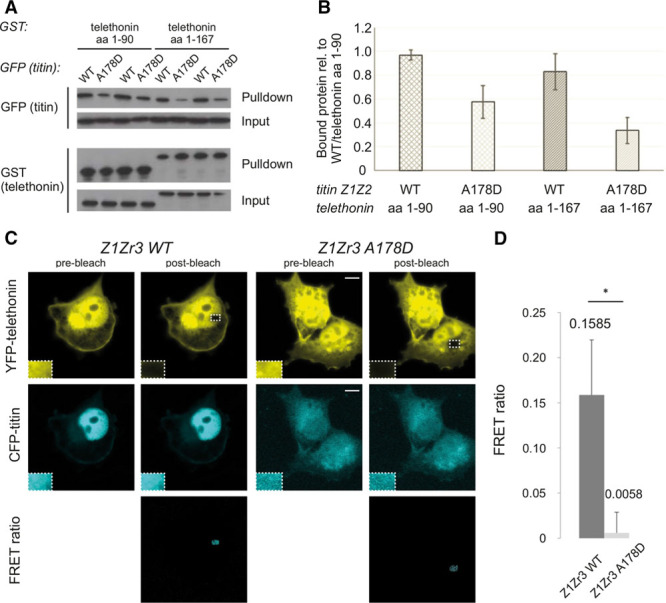
Functional implications of the titin Z1Z2 A178D mutation. **A**, Semiquantitative Glutathione S-transferase (GST) pulldown assays using telethonin fragments fused to GST (left aa 1–90, right full length) and titin Z1Z2 fragments (WT and A178D as indicated) as GFP fusions expressed in COS-1 cells. Bound titin-GFP fragments are detected by Western blotting (top row); input lysate controls are shown in the second row. Pulled down GST-telethonin fragments are shown in row three, as well as lysate controls (bottom row). **B**, Quantification of GST pulldown experiments from panel **A** visualizes reduced binding of titin Z1Z2 to telethonin in the presence of the A178D mutation; values are expressed as bound protein relative to lysate, with the first WT experiment set to 100% (n=2 per group, values expressed as mean with standard deviation for error bars; one representative experiment of 3 independent ones is shown). Because of the semiquantitative nature of the experiments, no statistical test was performed. **C**, Förster Resonance Energy Transfer (FRET) experiments using COS-1 cells cotransfected with telethonin (aa 1–90) fused to yellow fluorescent protein (YFP; first row) and titin Z1Zr3 fused to cyan fluorescent protein (CFP; second row). Images pre and post bleach are shown. FRET ratios are shown in the third row. Insets show magnification of indicated area. Scale bar represents 10 μm. **D**, Quantification of FRET efficiency for titin Z1Zr3 WT/A178D and telethonin pairs. The A178D mutation reduces the FRET efficiency from ≈0.15 to 0.01, indicating a dramatic loss of binding ability (WT n=20 and A178D n=26 cells; *P<0.0001 unpaired Student’s *t* test).

Localization of transfected Z1Z2 was not altered in the presence of the A178D mutation (Figure 4D). To assess the consequences of the mutation on binding telethonin, semiquantitative GST pulldown assays were performed with titin Z1Z2 and telethonin coexpressed in mammalian cells. Z1Z2 A178D showed impaired binding to 2 telethonin constructs (Figure 5A and 5B). The interaction between titin and telethonin was further quantified in Förster Resonance Energy Transfer experiments, where close proximity of proteins in a complex allows energy transfer from cyan fluorescent protein to yellow fluorescent protein between 2 fusion protein constructs.^31^ By introducing the A178D mutation into a Z1Zr3-cyan fluorescent protein construct, Förster Resonance Energy Transfer efficiency to telethonin-yellow fluorescent protein was almost abolished (Figure 5C and 5D), validating and quantifying the observation that A178D impairs binding to telethonin in the cellular context.

Taken together, our functional data suggest that the A178D mutant may affect protein folding and stability and impairs binding to telethonin, thus, supporting its pathogenic potential.

## Discussion

In this study, we present a 3-generation family with multiple individuals affected by cardiomyopathy with features of LVNC, systolic impairment, and an autosomal dominant inheritance pattern. Of note, the affected family members show a consistent phenotype with prominent hypertrabeculation as the main abnormality in the majority; this is relatively unusual because it is more typical to see LVNC in individual members of families with other forms of cardiomyopathy.

We used a combination of WGS in 2 affected individuals and linkage analysis in 13 family members; this approach identified only 2 rare candidate variants across the whole genome that segregated with the autosomal dominant cardiomyopathy. Because one of the identified genes (*PDP2*) is barely expressed in the heart, and the variant appears in implausible high numbers in the ExAC database, it is extremely unlikely to be disease causative. In contrast, titin, the gene affected by the other missense variant (*TTN* p.A178D), has crucial functions in the heart and is a known disease gene for cardiomyopathies (see below). Despite the fact that the family is too small for traditional genome-wide linkage analysis to identify the genetic cause of the disease (the logarithm of the odds score of 2.1, ie, odds ratio 1:125, is well below the threshold of 3.0, ie, odds ratio 1:1000), interrogation of the entire genome adds substantial weight to a likely causative role of the titin missense mutation for disease: no other plausible mutations, including larger genomic reorganizations (copy number variants), were detected in any other genes in the linkage regions, and the remainder of the genome is excluded by negative logarithm of the odds scores.

Titin has been implicated in cardiac and skeletal muscle disease, occasionally involving a combination of both. Mutations in this gene have been described in various forms of cardiomyopathy, such as DCM, arrhythmogenic right ventricular cardiomyopathy, hypertrophic cardiomyopathy, and restrictive cardiomyopathy (reviewed in Chauveau et al^27^). Truncating variants in titin (TTNtv) are the most frequent genetic finding in idiopathic DCM, being present in 15% to 25% of the cases,^32^ and are also frequent in peripartum cardiomyopathy (15%).^33^ However, penetrance seems to be low because TTNtv are also found in ≈1% of normal populations, and hence, the large majority of carriers do not manifest with disease.^34^ More recent work^35^ showed that DCM causing TTNtv are enriched in the sarcomeric A-band region, whereas TTNtv found in control cohorts tend to spare the A-band region and are in exons, with low usage in cardiac transcripts. An internal promotor in titin rescuing TTNtv N-terminally of the A-band region may explain this phenomenon.^36^

Titin missense mutations have been identified in DCM and hypertrophic cardiomyopathy cohorts.^4,37,38^ A causative role for *TTN* p. W976R in DCM is well supported by cosegregation within a large family and functional data.^39,40^ However, generally, titin missense mutations are challenging to interpret because rare benign variants are common in normal population cohorts. In the ExAC database, more than a third of the individuals carry a rare missense variant in titin (21 939 missense variants with <0.01% allelic frequency in 58 687 exomes), and although a proportion of these may represent recessive pathogenic alleles,^27^ only a small fraction will be disease-causing with dominant inheritance. Hence, clinical practitioners require cosegregation information to assign causality because bioinformatic prediction tools can only give probabilistic data.^4,37^ As we document here, interrogation of the entire genome combined with linkage analysis can help to narrow down lists of potential causative variants, even in small families.

Our finding of *TTN* p.A178D in a family with features of LVNC expands the spectrum of titinopathies: to our knowledge, this is the first report of a titin missense mutation implicated in cardiomyopathy with predominant features of LVNC and one of the first titin missense mutations supported by robust genome-wide genetics and detailed functional data. The latter suggests a likely pathogenic role of titin A178D by (1) evidence of protein degradation, partial unfolding, and domain destabilization in vitro, (2) protein destabilization in 2 cellular systems, and (3) altered binding properties to the ligand telethonin. Although extrapolations from such in vitro experiments on isolated domains to the full-length giant protein are not without uncertainty, such parameters will be useful complements in the future studies of other *TTN* missense variants. It is currently unclear how this particular mutation leads to this distinct phenotype, and more insight into the biology of Z-disk titin is needed to understand the underlying disease pathways. This will be addressed with the help of model organisms^36,41^ or patient-derived induced pluripotent stem cell–derived cardiomyocytes,^40^ focussing on the titin–telethonin complex^29^ and its downstream signaling targets^42^ in future work.

## Acknowledgments

We thank Stephan Lange (UCSD) for a titin Z1Z2 expression construct. Small-angle x ray scattering (SAXS) data were collected at the beamline P12, operated by EMBL, Hamburg unit, at the PETRA III storage ring (DESY, Hamburg, Germany). We gratefully thank Dmitry Svergun and his group for help with the SAXS data, the SPC facility at EMBL Hamburg for technical support, and Annabel Parret for her help with the Tridetector Analysis.

## Sources of Funding

Dr Gehmlich is supported by British Heart Foundation Grants (FS/12/40/29712, PG/15/113/31944). Drs Gehmlich, Hastings, and Watkins acknowledge support from the BHF Centre of Research Excellence, Oxford (grant codes HSRNWBY, HSRNWB11, and RE/13/1/30181). K.L. Thomson is the recipient of a National Institute for Health Research (NIHR) doctoral fellowship (NIHR-HCS-D13-04-006). This publication includes independent research supported also by the NIHR Biomedical Research Centre, Oxford. The work was supported also by funding from the Wellcome Trust Core Award Grant Number 090532/Z/09/Z. The views expressed are those of the authors and not necessarily those of the Department of Health or Wellcome Trust. Dr Gautel and A. Ghisleni were supported by the EU MUZIC network, the MRC, and the Leducq Foundation. Dr Gautel holds the BHF Chair of Molecular Cardiology.

## Disclosures

None.

## Supplementary Material

## Appendix

From the Division of Cardiovascular Medicine in the Radcliffe Department of Medicine, University of Oxford, and BHF Centre of Research Excellence, United Kingdom (R.H., C.P.d.V., C.H., L.O., K.L.T., L.A., H.W., K.G.); NIHR Biomedical Research Centre Oxford (A.P., S.J.L.K., J.C.T.) and Wellcome Trust Centre for Human Genetics (A.P., S.L., S.S., S.J.L.K., J.C.T.), University of Oxford, United Kingdom; Department of Clinical Genetics, Churchill Hospital, Oxford University NHS Trust, Oxford, United Kingdom (K.L.T., E.B.); European Molecular Biology Laboratory, Hamburg, Germany (S.D.C., P.V.K., M.W.); Laboratory of Reflectometry and Small-Angle Scattering, A.V. Shubnikov Institute of Crystallography, Russian Academy of Sciences, Moscow, Russian Federation (P.V.K.); and Randall Division of Cell and Molecular Biophysics and Cardiovascular Division, King’s College London BHF Centre of Research Excellence, London, United Kingdom (E.E., A.G., M.G.).

## CLINICAL PERSPECTIVE

High throughput next-generation sequencing techniques have made whole genome sequencing accessible and are increasingly applied in clinical practice. However, the abundance of variation in the human genomes makes the identification of a disease-causing mutation on a background of benign rare variants challenging. To illustrate, more than one third of individuals in normal population cohorts carry a rare missense variant in the giant protein titin (coded by the gene *TTN*), but only a small fraction of these will be disease-causing with dominant inheritance. Hence, titin missense variants are currently often ignored or left uninterpreted when found in cardiomyopathy patients. Here we combine whole genome sequencing with linkage analysis in a 3-generation family affected by cardiomyopathy with features of autosomal dominant left ventricular noncompaction cardiomyopathy. A missense mutation in titin (*TTN* p. A178D) is the only plausible disease-causing variant that segregates with disease among affected individuals of the family, with interrogation of the entire genome excluding other potential causes. Functional studies on this missense mutation demonstrate domain misfolding and destabilization, resulting in paired binding to the ligand telethonin/t-cap and, hence, supporting its highly likely causative role. Our report expands the spectrum of titin’s roles in cardiomyopathies and furthermore highlights that rare titin missense variants should be considered to be relevant for cardiomyopathies and can be identified by combining whole genome sequencing with linkage analysis in medium-sized cardiomyopathy families.
