# Supplementary material for: Combination of Whole Genome Sequencing, Linkage, and Functional Studies Implicates a Missense Mutation in Titin as a Cause of Autosomal Dominant Cardiomyopathy With Features of Left Ventricular Noncompaction
Source: Circ Cardiovasc Genet. 2016 Oct 18;9(5):426–35. doi: 10.1161/CIRCGENETICS.116.001431 (PMC5068189; doi:10.1161/CIRCGENETICS.116.001431)
Supplement: Supplementary file 1 [file hcg-9-426-s001.pdf]

Supplemental Material

Expanded Methods

Copy Number Analyses

Copy number analyses were carried out for all family members (see Figure 1A) except II-4 and III-2 (for whom DNA was unavailable). For each individual, ~200ng DNA was tested against a sub-array of an Illumina HumanCytoSNP-12v1 BeadChip (Illumina Inc, San Diego, CA) according to manufacturer's guidelines. Data analyses were performed with GenomeStudio v2009.2 (Illumina Inc., San Diego, CA) and finalreport.txt files uploaded into Nexus Copy Number v7.5.3 Discovery Edition (BioDiscovery, Hawthorne, CA) for visualisation and evaluation of copy number (CN) events, looking for events that that might segregate with and contribute to affected cardiomyopathy status as given in the pedigree (Figure 1A). In addition, WGS data (.bam files) from the two first cousins (III-1 and III-4) were processed using ngbin (https://github.com/) set with a window size of 1000 and read count filter of 10. The ngbin output was uploaded into Nexus Copy Number v7.5.3 Discovery Edition for visualisation and CN events evaluated and noted. In the WGS data, centromeric and telomeric peaks and peaks mapping to SD sequences were excluded. CN events were not considered relevant with respect to the cardiomyopathy phenotype if they had been noted previously at >1% frequency in unrelated datasets (Database of Genomic Variants http://dgv.tcag.ca/dgv/app/home or 'in house') or did not co-segregate in family members with a confirmed cardiomyopathy (see Figure 1).

LOD score calculation

A standard disease model for rare dominant conditions was used to calculate the LOD score: allele frequency = 0.0001 (0.01%) and probability of being affected for individuals with 0, 1 and 2 copies of the disease allele (penetrance) was 0.0001, 1.0 and 1.0 respectively (i.e. complete penetrance for heterozygotes) on Merlin (version 1.1.2) software.

Expression profiles of genes

Evidence of expression of the genes in the heart both at RNA and protein level was interrogated in following databases:

GeneCards	http://www.genecards.org/
Expression Atlas (EMBL-EBI)	https://www.ebi.ac.uk/gxa/home
The Human Protein Atlas	http://www.proteinatlas.org/
ProteomicsDB	https://www.proteomicsdb.org/
PaxDB	http://pax-db.org/#!home
GTex Portal	http://www.gtexportal.org/home/
GeneHub	http://share.gene.com/Research/ genentech/genehub-gepis/index.html

Biophysical characterisation of titin Z1Z2 protein fragments

Size Exclusion Chromatography-Tridetector Analysis: The two proteins, WT Z1Z2 and the A178D mutant were analysed with a Viscotek 305 tridetector (Malvern Instruments, Malvern, UK), equipped with light scattering, refractive index, and UV absorbance detectors. Malvern was connected to an analytical size exclusion column (Superdex 200 10/300 GL; GE Healthcare), at a flow rate of 0.3 mL/min, using 25 mm Tris (pH 8.0), 200 mm NaCl as running buffer. The protein

concentration was 3.6 and 3.8 mg/mL for the WT and A178D respectively and the sample volume was 100 µl. The refractive index combined with light-scattering data were used for the estimation of the molecular masses. BSA, re-suspended in the same buffer, was used as internal control.

Circular Dichroism (CD) spectropolarimetry measurements: Prior to each measurement, the two samples were dialyzed against 10 mM potassium phosphate, pH 7.5, 100 mM NaF and diluted to 10 µM. The spectra were recorded on a Chirascan CD Spectrometer (Applied Photophysics), between 190 and 260 nm, at 10°C, using a 0.5 mm cuvette. The background was subtracted and each spectrum was converted to mean residue ellipticity. Each curve represents the average of three measurements.

Thermal stability assay: The thermal stability of the two variants was assessed by differential scanning fluorimetry (Thermofluor™), using a iCycleMyIQ RT-PCR Detection System (Bio-Rad), equipped with a CCD detector for imaging of the fluorescence. SYPRO Orange was added as a fluorophore. The protein was concentrated at 50 and 100 µM, in a buffer containing 25 mm Tris (pH 8.0) and 200 mm NaCl. The plate was heated from 5 to 95 °C with initial stepwise increments of 1 °C per minute. Each curve represents the average of five separate measurements.

SAXS measurements and data processing: Synchrotron radiation X-ray scattering data were collected on the EMBL P12 beamline at the PETRA III storage ring (DESY, Hamburg). Solutions of Z1Z2 WT and A178D mutant were measured at 10 °C at solute concentrations of 2.7, 5,6 and 10.3 mg/ml. PILATUS 2M detector (Dectris, Switzerland) was used at the sample-detector distance 3.0 m and wavelength λ=0.1 nm, covering the momentum transfer range $0.05 < s < 4.5$ nm^{-1} (s

= $4\pi \sin\theta/\lambda$, where 2θ is the scattering angle). The samples had no measurable radiation damage detected by comparison of twenty successive time frames with 50 ms exposures. The data were averaged after normalization to the intensity of the transmitted beam and the scattering of the buffer was subtracted. The difference data were extrapolated to zero solute concentration following standard procedures. All data manipulations were performed using the program package PRIMUS [1].

The radius of gyration R_g of solutes and the forward scattering $I(0)$ were evaluated using the Guinier approximation at small angles ($s < 1.3/R_g$) [2] (assuming that the intensity is represented as $I(s) = I(0)\ exp(-(sR_g)^2/3)$ and also from the entire scattering pattern by the program GNOM [3]. In the latter case the distance distribution functions $p(r)$ and the maximum particle dimensions D_{max} were also computed. The molecular masses (MM) of the solutes were evaluated by comparison of the calculated $I(0)$ value with that of the standard solution of bovine serum albumin (MM 66 kDa).

The excluded volumes of hydrated protein molecules (V_p) were calculated using the Porod approximation:

$$V_p = 2\pi^2 I(0) / \int_0^{\infty} s^2 I_{\exp}(s) ds \qquad (1)$$

in which the intensity $I(s)$ was modified by subtraction of an appropriate constant from each data point to force the s^{-4} decay of the intensity at higher angles following the Porod's law [4] for homogeneous particles.

Low resolution *ab initio* model of Z1Z2 WT and A178D mutant were generated by the program DAMMIF [5], which represents the protein by an assembly of densely packed beads. Simulated annealing (SA) was employed to build a compact

interconnected configuration of beads that fits the experimental data $I_{exp}(s)$ to minimize the discrepancy:

$$\chi^2 = \frac{1}{N-1}\sum_j \left[\frac{I(s_j) - cI_{calc}(s_j)}{\sigma(s_j)} \right]^2 \tag{2}$$

where N is the number of experimental points, c is a scaling factor, $I_{calc}(s_j)$ and $\sigma(s_j)$ are the calculated intensity and the experimental error at the momentum transfer s_j, respectively. The common structural features of the model were determined by superimposing and averaging of the configurations from ten separate runs using the programs SUPCOMB [6] and DAMAVER [7].

The scattering pattern of the crystallographic coordinates of wild-type Z1Z2 (PDB code: 2A38) was calculated using CRYSOL [8]. Given the atomic coordinates, the program fits the experimental intensity by adjusting the excluded volume of the particle and the contrast of the hydration layer to minimize the discrepancy, defined by χ^2 (equation 2).

Foerster Resonance Energy Transfer Experiments

WT human titin Z1Zr3 was cloned in the expression vector pECFP-C1 between the XhoI and BamHI restriction sites, with an N-terminal CFP-tag. The same plasmid was used for site-directed mutagenesis of the titin Z1Zr3 A178D construct. Wild-type human telethoninΔC (residues 1-90) was cloned in the expression vector pEYFP-C1 between the XhoI and BamHI restriction sites, with N-terminal YFP-tag. Sequences were validated by DNA sequencing.

COS-1 cells were cultured in Dulbecco's modified Eagle medium supplemented with 10% fetal bovine serum and 100 U/mL penicillin/streptomycin (Invitrogen Life Technologies). For expression and microscopic analysis of

fluorescent fusion proteins, cells were grown on plastic slides and transfected with appropriate plasmid DNA using Escort IV transfection reagent (Sigma-Aldrich, USA) according to the manufacturer's instructions. Cells were fixed for 10 min with 4% paraformaldehyde 24–36 h post-transfection and specimens were mounted in anti-fade medium.

To detect FRET by the acceptor photobleaching method, cells transfected with appropriate combinations of donor and acceptor expression constructs were imaged on a Zeiss LSM 510 confocal microscope using a 63X 1.4NA Plan NeoFluar oil immersion objective. CFP was excited with the 458 nm line of an Argon-Krypton ion laser and YFP was excited using the 514 nm argon line, respectively. A 545 nm dichroic mirror was used to split the two emission channels, followed by a band-pass 475–525 nm filter for the CFP channel and a long pass 530 nm filter for the YFP channel. The coverslip was first scanned at 10x magnification, marking cells showing strong comparable signals for both CFP and YFP channels. During preliminary scans with the 63X Objective, a rectangular acquisition Region of Interest (ROI) was chosen where homogeneous signals for each channel were detected. The gain for the CFP and YFP channels were set to the nearest grey-value to maximum without saturating any pixels, at approximately 75% of the dynamic range (12-bit, 4096 grey levels), with offsets set such that backgrounds were zero. Any saturated pixels were automatically excluded from FRET calculations in order to not affect FRET results. Time-lapse mode was used to collect one pre-bleach image for each channel before bleaching. YFP was photobleached using the 514 nm line at maximum power for 150 iterations. A second post-bleach image was then collected for each channel. Pre and post-bleach CFP and YFP images were processed using the software Image-J (NIH) and user-written Macros. Images were background-subtracted using rolling ball

radius (sliding paraboloid, 3.0-5.0 pixels radius), and pre- and post-bleach images fade-corrected. FRET efficiency (E_r) was calculated according to equation (3):

$$E_r = (CFP_{postbleach} - CFP_{prebleach}) / CFP_{postbleach} \qquad (3)$$

By using the equation (4):

$$E_{ext} = E_r / Bleach_{efficiency} \qquad (4)$$

FRET efficiency can be extrapolated to theoretical bleach efficiency 100% (E_{ext}) according to the linear correlation previously described [9]. Mean values from three independent cell transfections were plotted for comparison. Error bars represent standard deviations. Unpaired student's test was performed and $p < 0.05$ was considered significant.

Assessment of protein stability

Human Titin Z1Z2 WT and A178D were cloned into the pShuttle-hrGFP-2 vector (Agilent). Adenoviral particles were generated, scaled up and purified using the AdEasy system (Agilent) according to the manufacturer's instructions. NRC were infected at MOI = 5 and harvested after 48 hrs. Total protein extracts were generated and assayed by Western blotting as described [10] using anti-HA monoclonal rat antibody (Roche), anti-hrGFP polyclonal rabbit antibody (Agilent) and anti-GAPDH polyclonal rabbit antibody (Millipore). Western blots were quantified using ImageLab software (Biorad), intensity normalised to loading control and expressed relative to Z1Z2 WT (set to 100 %). RNA was isolated from identically infected NRC using the RNeasy Mini kit (Qiagen) and transcribed with High Capacity cDNA Reverse transcription Kit (Applied Biosystems) prior to amplification reactions performed using Fast Universal Master Mix on a StepOnePlus system (Applied Biosystems). Samples were run in duplicate in a total reaction volume of 10 µL. Following TaqMan

assays were used: human recombinant titin (not detecting rat *Ttn*): Hs01562028_m1, rat *Gapdh*: Rn99999916_s1 (both inventoried), hrGFP – assay ID AIY9YGZ (custom designed). Relative expression was quantified using the comparative CT method.

COS-1 cells were cultured and transfected as for the FRET experiments using HA-tagged constructs coding for human titin Z1Z2 WT or A178D (1 mug DNA for WT and 2.5 mug for A178D, respectively). Cells were treated with translational inhibitor cycloheximide at 20 mug/ml for 8 and 24 hrs, vehicle control (DMSO) was applied for 24 hrs. Total protein extracts were prepared and blotted for HA-tag as above and beta-actin as loading control (polyclonal rabbit antibody, Sigma). Western blots were quantified using ImageLab software (Biorad), intensity normalised to loading control and expressed relative to vehicle control values (set to 100 %).

Additional Figures

Figure S1: Cardiac MRI images demonstrating prominent features of LVNC in affected individuals III-3, III-4 and III-5, but not III-6. II-4 has a dilated LV. End-diastolic four chamber views are shown, numbering of individuals as in Figure 1A and Table 1.

Figure S2: Linkage plots for each chromosome from SNP array data analysed in MERLIN software. Red lines indicate a LOD score of 0 and the approximate positions of *TTN* and *PDP2* are indicated by arrows.

Figure S3: A – Comparison of DNA sequence between a synthetically derived reference sequence (top panel) and individual II-4 (middle panel). The heterozygous missense variant is indicated by the black arrow. The bottom panel illustrates the signal-to-noise ratio between the reference sequence and the patient sequence. The presence of the heterozygous single nucleotide variant *TTN* c.533C>A (*TTN* p. A178D) is clearly distinguishable (raised peak) from the background noise (using Mutation Surveyor software, Softgenetics).

B – Structural alignment using deposited structures of titin Ig-domains. The position of beta-strands A to G is marked and the position of A178 indicated with an asterisk. Apart from Alanine, position 178 is exclusively occupied by hydrophobic side chains (Val, Leu, Ile) or cysteine. Structural alignment was performed by PDBeFold (SSM) [11] using secondary structure matching. Sequence conservation analysis was done by the ConSruf server [12].

Figure S4 – Thermal denaturation experiments: Titin Z1Z2 WT (solid line) unfolds with a melting temperature of approximately 62 degrees. Titin Z1Z2 A178D (broken line) displays high fluorescence signal already at low temperatures, and no melting temperature can be deducted for Titin Z1Z2 A178D.

Figure S5 SAXS measurements: A – Normalized Kratky plot displays a bell-shaped profile for Z1Z2 WT (1, black symbols) typical for folded structures, whereas for the A178D mutant (2, white symbols) it appears to be intermediate between folded and completely unfolded structures [13] suggesting the presence of unfolded parts/flexible domains in the A178D mutant. B – Scattering patterns from Z1Z2 WT (1) and Z1Z2 A178D (2) and their models: experimental data (WT in black and A178D in white dots) are shown with error bars representing one standard deviation; scattering computed from *ab initio* models are plotted with solid lines, scattering from the monomeric Z1Z2 WT model (PDB code: 2A38) with dashed lines. Insert: Distance distribution functions *p(r)* for Z1Z2 WT (black symbols) and for Z1Z2 A178D (white symbols). The asymmetric tails of the distance distribution functions for both Z1Z2 WT and Z1Z2 A178D are consistent with an elongated shape of the proteins.

Figure S6 A – Control experiments to demonstrate equal MOIs for WT and A178D in experiments shown in Figure 4B. Quantification of Western blots from Figure 4C is shown in grey bars. For mRNA measurements (black bars), NRCs were transfected as in Figure 4C and analysed RNA quantified by TaqMan assays for human titin Z1Z2 and the hrGFP reporter. B, C – Pulse chase experiments confirming reduced stability of titin Z1Z2 A178D protein fragment in COS-1 cells. B – Cells were transfected with constructs coding for HA-tagged titin Z1Z2 WT (left) or titin Z1Z2

A178D (right) mutant protein fragment and treated with cycloheximide (CHX) to stop protein translation for 8 and 24 hours. Vehicle treated cells (24 hrs) served as control. Expression of HA-tagged titin Z1Z2 was assayed from total protein lysates by Western blotting for HA-tag, probing for endogenous beta-actin served as loading control. C – Quantification of HA-tagged titin Z1Z2 normalised to beta-actin: Amounts are shown relative to vehicle control (set to 100 %). Titin Z1Z2 WT fragment (grey bars) is more stable than titin Z1Z2 A178D mutant protein fragment (black bars).

Figure S7. NRC were transfected with titin Z1Z2 WT or A178D as in Figure 4D, but counterstained for endogenous telethonin. Merged images are shown in the third row, HA shown in red, endogenous telethonin in green. No changes in localisation are observed for telethonin in the presence of titin Z1Z2 A178D. Scale bar represents 10 microns.

Additional Tables

Table S1: Filtering criteria applied to all variants identified.

Table S2: Variants remaining after automated filtering.

Table S3: Analysis and exclusion of an intronic/ splice region variant in titin.

Table S4: Primer sequences.

Supplemental References

1. Konarev PV, Volkov VV, Sokolova AV, Koch MHJ, Svergun DI. PRIMUS: a Windows PC-based system for small-angle scattering data analysis. *J Appl Crystallogr*. 2003;36:1277-1282.

2. Guinier A. La diffraction des rayons X aux tres petits angles; application a l'etude de phenomenes ultramicroscopiques. *Ann Phys (Paris)* 1939;12:161-237.

3. Svergun D. Determination of the regularization parameter in indirect-transform methods using perceptual criteria. *J Appl Crystallogr.* 1992;25:495-503.

4. Porod G. General theory. In: O. K. Glatter, O., ed. *Small-angle X-ray scattering* London: Academic Press; 1982.

5. Franke D, Svergun DI. DAMMIF, a program for rapid ab-initio shape determination in small-angle scattering. *J Appl Crystallogr.* 2009;42:342-346.

6. Kozin MB, Svergun DI. Automated matching of high- and low-resolution structural models. *J Appl Crystallogr.* 2001;34:33-41.

7. Volkov VV, Svergun DI. Uniqueness of ab initio shape determination in small-angle scattering. *J Appl Crystallogr.* 2003;36:860-864.

8. Svergun D, Barberato C, Koch MHJ. CRYSOL - a Program to Evaluate X-ray Solution Scattering of Biological Macromolecules from Atomic Coordinates. *J Appl Crystallogr.* 1995;28:768-773.

9. Menon RP, Soong D, de Chiara C, Holt MR, Anilkumar N, Pastore A. The importance of serine 776 in Ataxin-1 partner selection: a FRET analysis. *Sci Rep.* 2012;2:919.

10. Geier C, Gehmlich K, Ehler E, Hassfeld S, Perrot A, Hayess K , et al. Beyond the sarcomere: CSRP3 mutations cause hypertrophic cardiomyopathy. *Hum Mol Genet*. 2008;17:2753-2765.

11. Krissinel E, Henrick K. Inference of macromolecular assemblies from crystalline state. *J Mol Biol*. 2007;372:774-797.

12. Celniker G, Nimrod G, Ashkenazy H, Glaser F, Martz E, Mayrose I , et al. ConSurf: Using Evolutionary Data to Raise Testable Hypotheses about Protein Function. *Isr J Chem*. 2013;53:199-206.

13. Receveur-Brechot V, Durand D. How random are intrinsically disordered proteins? A small angle scattering perspective. *Curr Protein Pept Sci*. 2012;13:55-75.

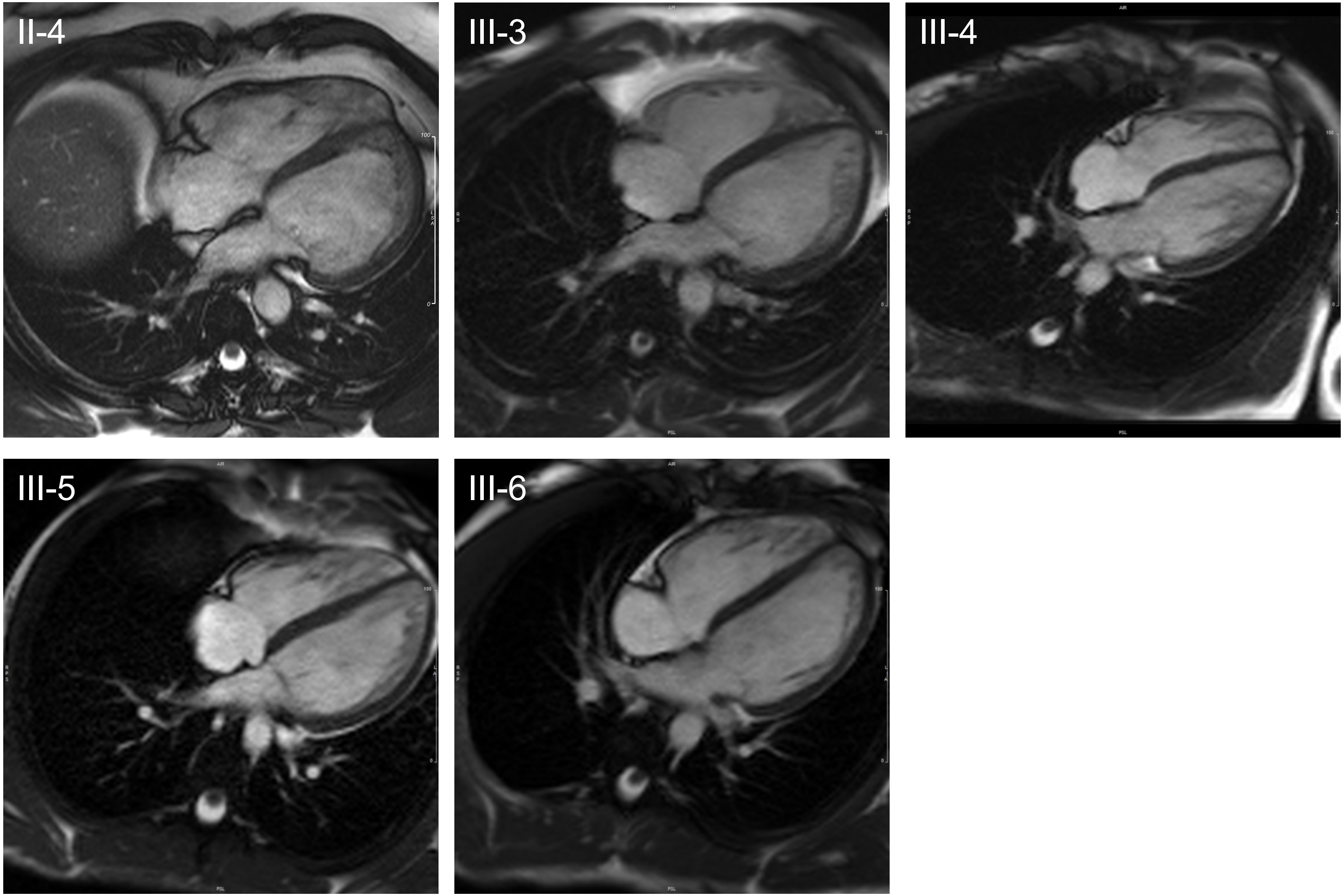

Figure S1

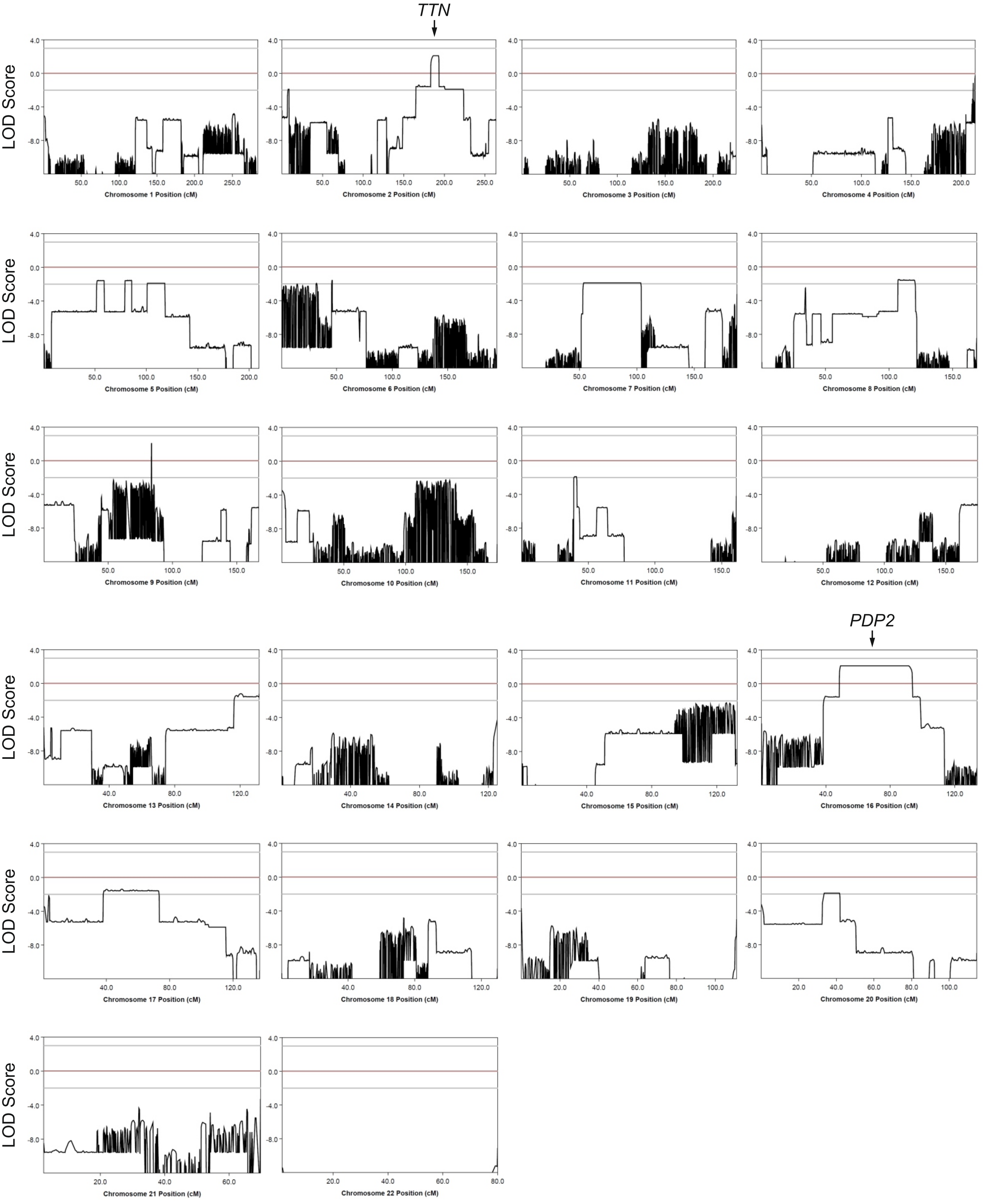
TTN
PDP2
LOD Score
4.0
0.0
-4.0
-8.0
Chromosome 1 Position (cM)
Chromosome 2 Position (cM)
Chromosome 3 Position (cM)
Chromosome 4 Position (cM)
Chromosome 5 Position (cM)
Chromosome 6 Position (cM)
Chromosome 7 Position (cM)
Chromosome 8 Position (cM)
Chromosome 9 Position (cM)
Chromosome 10 Position (cM)
Chromosome 11 Position (cM)
Chromosome 12 Position (cM)
Chromosome 13 Position (cM)
Chromosome 14 Position (cM)
Chromosome 15 Position (cM)
Chromosome 16 Position (cM)
Chromosome 17 Position (cM)
Chromosome 18 Position (cM)
Chromosome 19 Position (cM)
Chromosome 20 Position (cM)
Chromosome 21 Position (cM)
Chromosome 22 Position (cM)

Figure S2

A

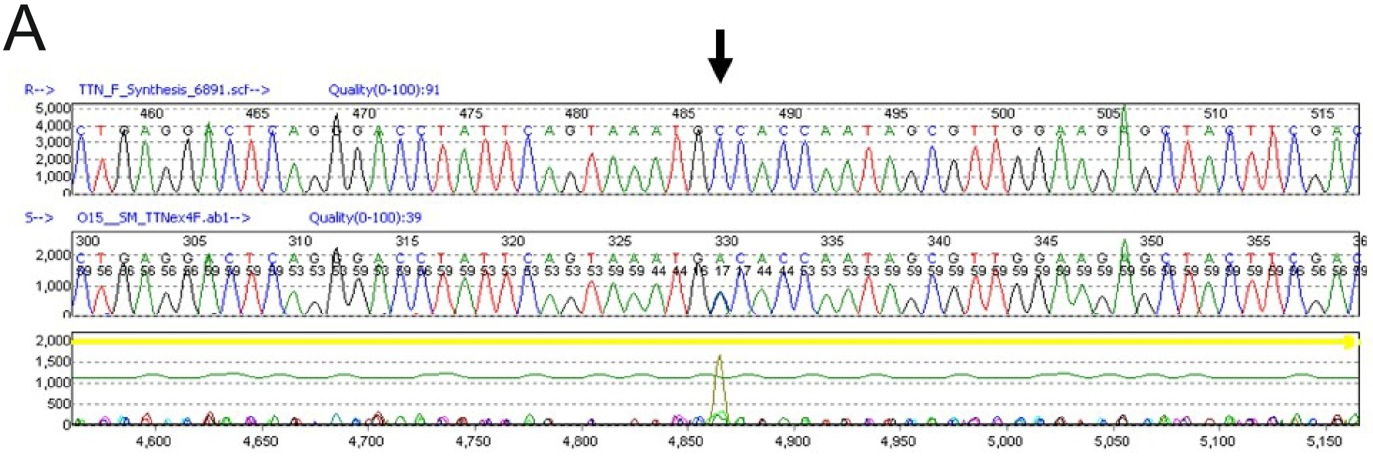
R--> TTN_F_Synthesis_6891.scf--> Quality(0-100):91
S--> O15__SM_TTNex4F.ab1--> Quality(0-100):39

B

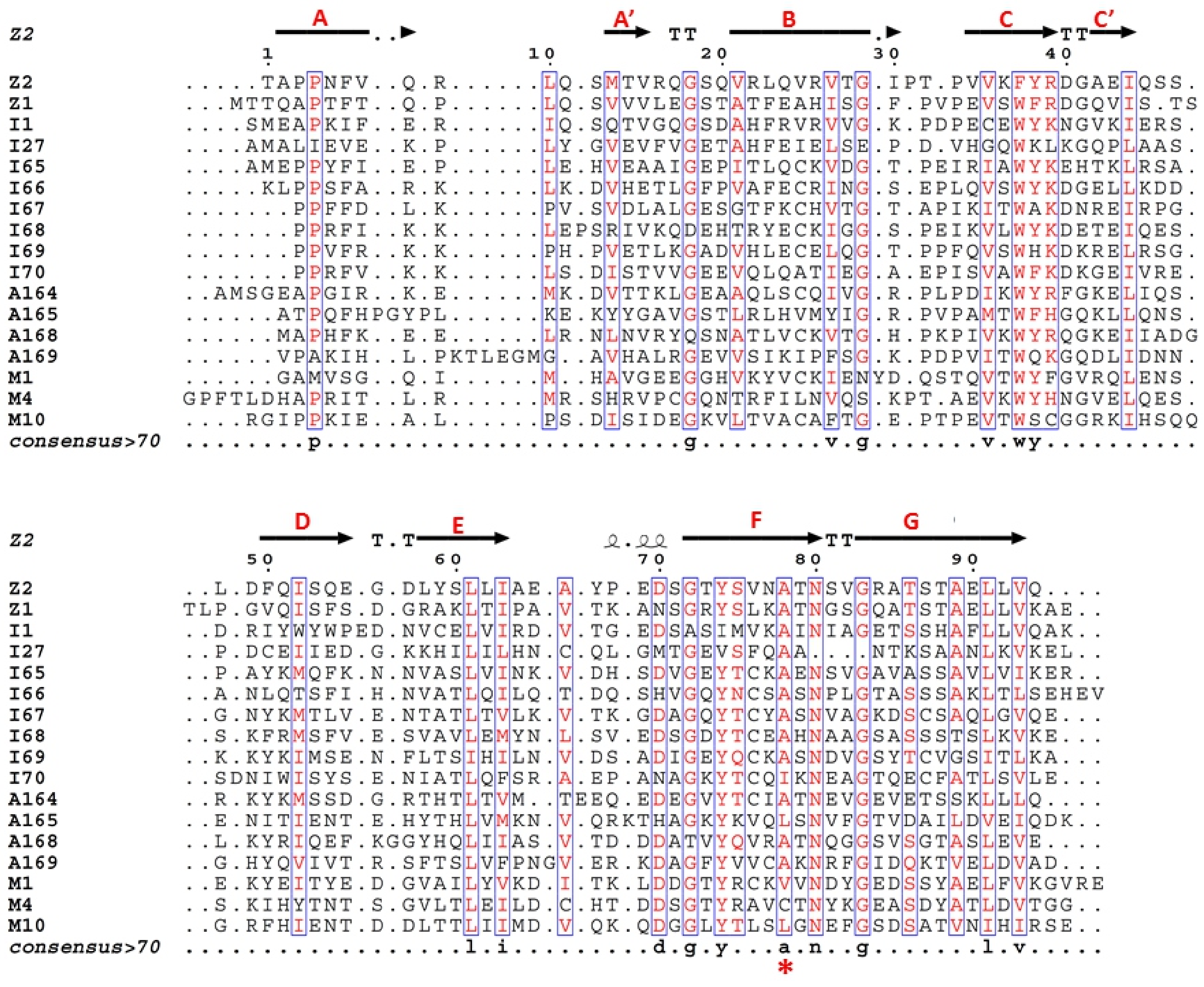
A
A'
B
C
C'
Z2
Z2 .....TAPPNFV..Q.R......LQ.SMTVRQGSQVRLQVRVTG.IPT.PVVKFYRDGAEIQSS.
Z1 ...MTTQAPTFT..Q.P......LQ.SVVVLEGSTATFEAHISG.F.PVPEVSWFRDGQVIS.TS
I1 ....SMEAPKIF..E.R......IQ.SQTVGQGSDAHFRVRVVG.K.PDPECEWYKNGVKIERS.
I27 ....AMALIEVE..K.P......LY.GVEVFVGETAHFEIELSE.P.D.VHGQWKLKGQPLAAS.
I65 ....AMEPPYFI..E.P......LE.HVEAAIGEPITLQCKVDG.T.PEIRIAWYKEHTKLRSA.
I66 .....KLPPSFA..R.K......LK.DVHETLGFPVAFECRING.S.EPLQVSWYKDGELLKDD.
I67 .......PPFFD..L.K......PV.SVDLALGESGTFKCHVTG.T.APIKITWAKDNREIRPG.
I68 .......PPRFI..K.K......LEPSRIVKQDEHTRYECKIGG.S.PEIKVLWYKDETEIQES.
I69 .......PPVFR..K.K......PH.PVETLKGADVHLECELQG.T.PPFQVSWHKDKRELRSG.
I70 .......PPRFV..K.K......LS.DISTVVGEEVQLQATIEG.A.EPISVAWFKDKGEIVRE.
A164 ..AMSGEAPGIR..K.E......MK.DVTTKLGEAAQLSCQIVG.R.PLPDIKWYRFGKELIQS.
A165 ......ATPQFHPGYPL......KE.KYYGAVGSTLRLHVMYIG.R.PVPAMTWFHGQKLLQNS.
A168 ......MAPHFK..E.E......LR.NLNVRYQSNATLVCKVTG.H.PKPIVKWYRQGKEIIADG
A169 ......VPAKIH..L.PKTLEGMG..AVHALRGEVVSIKIPFSG.K.PDPVITWQKGQDLIDNN.
M1 ......GAMVSG..Q.I......M..HAVGEEGGHVKYVCKIENYD.QSTQVTWYFGVRQLENS.
M4 GPFTLDHAPRIT..L.R......MR.SHRVPCGQNTRFILNVQS.KPT.AEVKWYHNGVELQES.
M10 ....RGIPPKIE..A.L......PS.DISIDEGKVLTVACAFTG.E.PTPEVTWSCGGRKIHSQQ
consensus>70 ....p....................g........v.g.......v.wy..........
D
E
F
G
Z2
Z2 ..L.DFQISQE.G.DLYSLLIAE.A.YP.EDSGTYSVNATNSVGRATSTAELLVQ....
Z1 TLP.GVQISFS.D.GRAKLTIPA.V.TK.ANSGRYSLKATNGSGQATSTAELLVKAE..
I1 ..D.RIYWYWPED.NVCELVIRD.V.TG.EDSASIMVKAINIAGETSSHAFLLVQAK..
I27 ..P.DCEIIED.G.KKHILILHN.C.QL.GMTGEVSFQAA....NTKSAANLKVKEL..
I65 ..P.AYKMQFK.N.NVASLVINK.V.DH.SDVGEYTCKAENSVGAVASSAVLVIKER..
I66 ..A.NLQTSFI.H.NVATLQILQ.T.DQ.SHVGQYNCSASNPLGTASSSAKLTLSEHEV
I67 ..G.NYKMTLV.E.NTATLTVLK.V.TK.GDAGQYTCYASNVAGKDSCSAQLGVQE...
I68 ..S.KFRMSFV.E.SVAVLEMYN.L.SV.EDSGDYTCEAHNAAGSASSSTSLKVKE...
I69 ..K.KYKIMSE.N.FLTSIHILN.V.DS.ADIGEYQCKASNDVGSYTCVGSITLKA...
I70 ..SDNIWISYS.E.NIATLQFSR.A.EP.ANAGKYTCQIKNEAGTQECFATLSVLE...
A164 ..R.KYKMSSD.G.RTHTLTVM..TEEQ.EDEGVYTCIATNEVGEVETSSKLLLQ....
A165 ..E.NITIENT.E.HYTHLVMKN.V.QRKTHAGKYKVQLSNVFGTVDAILDVEIQDK..
A168 ..L.KYRIQEF.KGGYHQLIIAS.V.TD.DDATVYQVRATNQGGSVSGTASLEVE....
A169 ..G.HYQVIVT.R.SFTSLVFPNGV.ER.KDAGFYVVCAKNRFGIDQKTVELDVAD...
M1 ..E.KYEITYE.D.GVAILYVKD.I.TK.LDDGTYRCKVVNDYGEDSSYAELFVKGVRE
M4 ..S.KIHYTNT.S.GVLTLEILD.C.HT.DDSGTYRAVCTNYKGEASDYATLDVTGG..
M10 ..G.RFHIENT.D.DLTTLIIMD.V.QK.QDGGLYTLSLGNEFGSDSATVNIHIRSE..
consensus>70 ....l.i.........d.g.y...a.n.g.......l.v.....

Figure S3

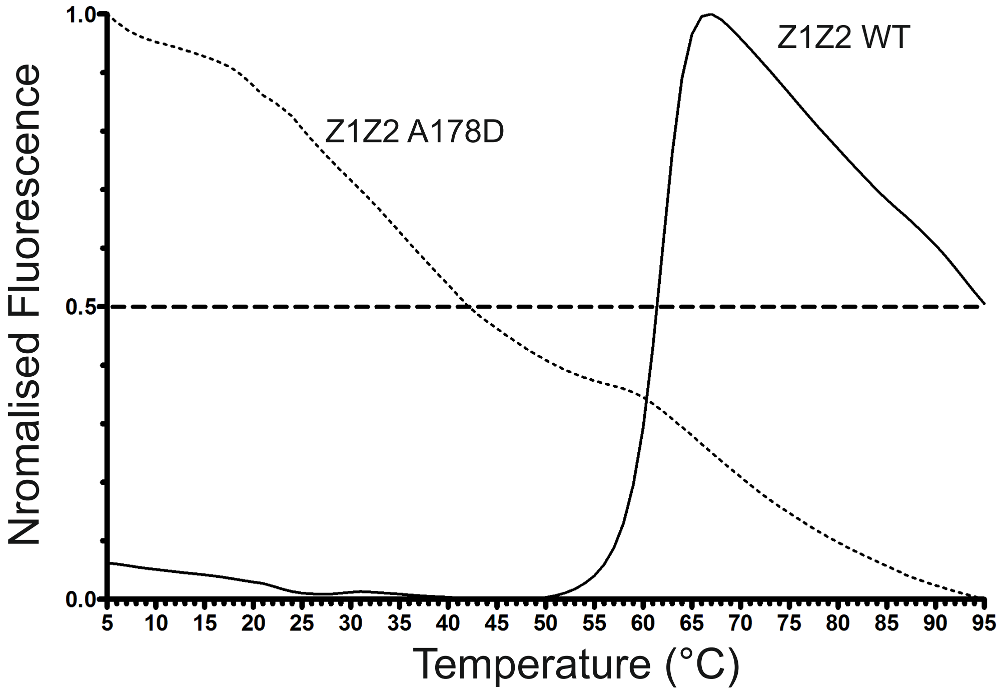
1.0
0.5
0.0
Nromalised Fluorescence
Z1Z2 A178D
Z1Z2 WT
5 10 15 20 25 30 35 40 45 50 55 60 65 70 75 80 85 90 95
Temperature (°C)

Figure S4

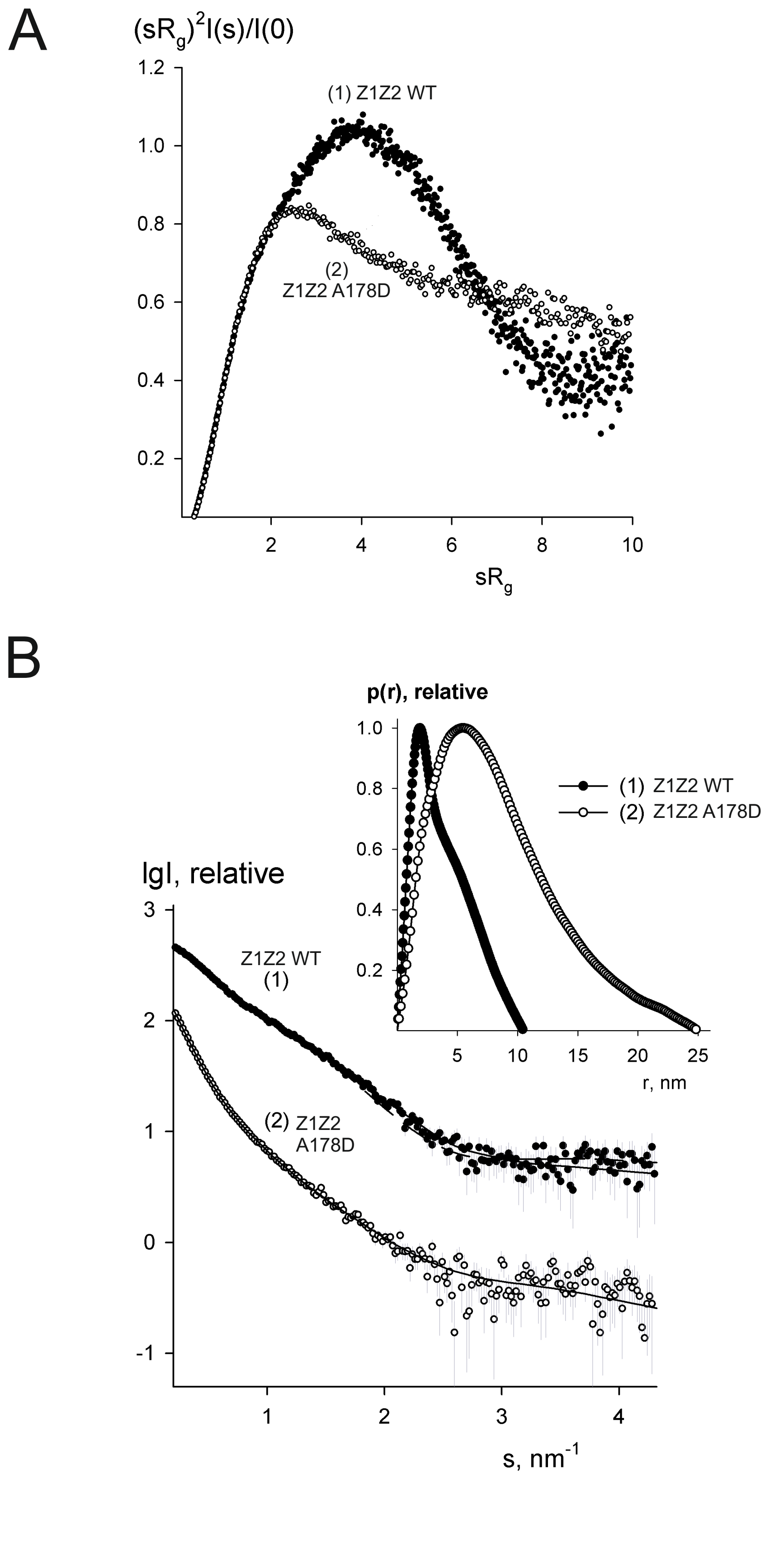

A
(sR$_g$)^{2}I(s)/I(0)
1.2
1.0
0.8
0.6
0.4
0.2
(1) Z1Z2 WT
(2)
Z1Z2 A178D
2
4
6
8
10
sR$_g$
B
p(r), relative
1.0
0.8
0.6
0.4
0.2
5
10
15
20
25
r, nm
(1) Z1Z2 WT
(2) Z1Z2 A178D
lgI, relative
3
2
1
0
-1
Z1Z2 WT
(1)
(2) Z1Z2
A178D
1
2
3
4
s, nm^{-1}

Figure S5

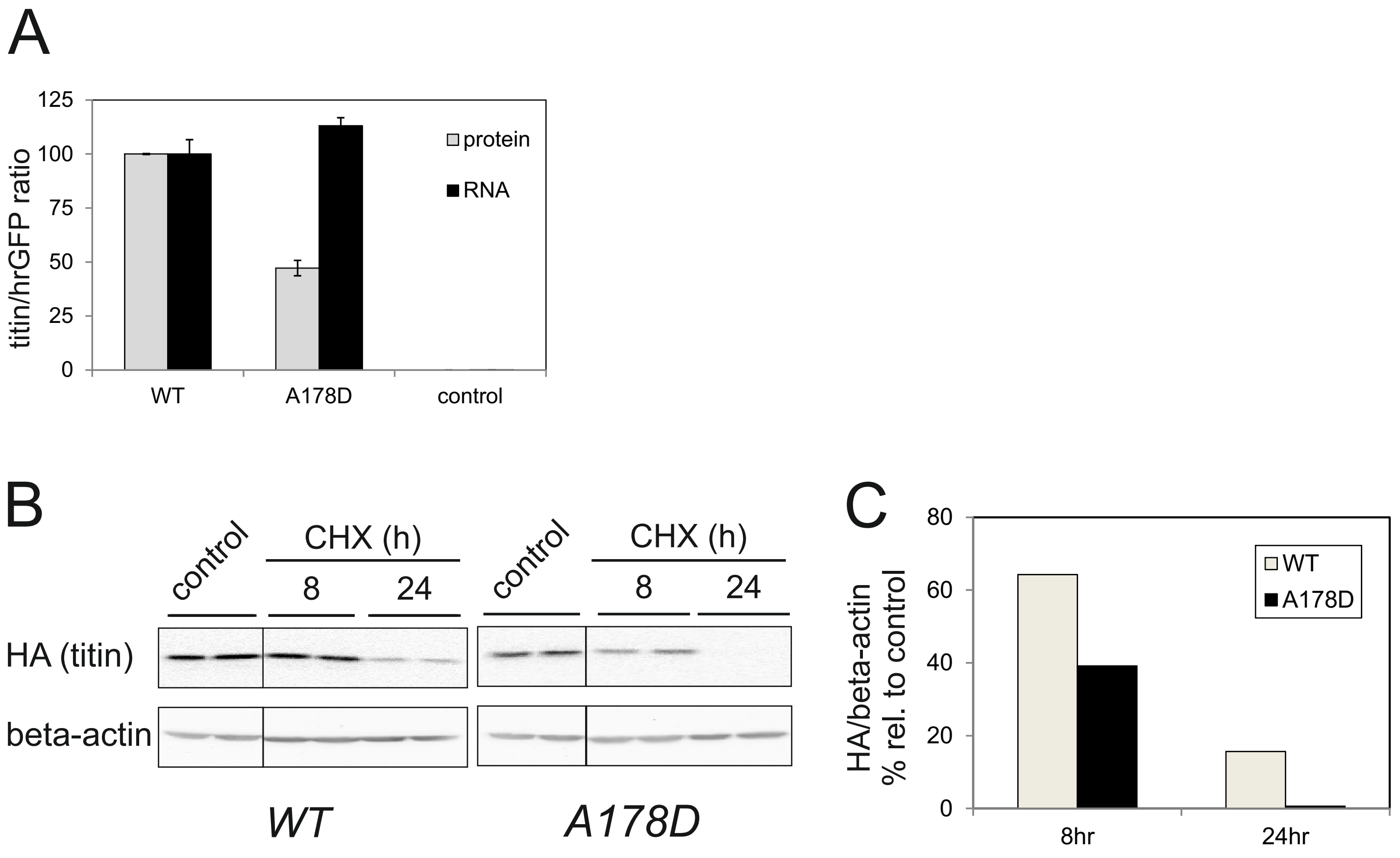
A
titin/hrGFP ratio
125
100
75
50
25
0
protein
RNA
WT
A178D
control
B
control
CHX (h)
8
24
control
CHX (h)
8
24
HA (titin)
beta-actin
WT
A178D
C
HA/beta-actin
% rel. to control
80
60
40
20
0
WT
A178D
8hr
24hr

Figure S6

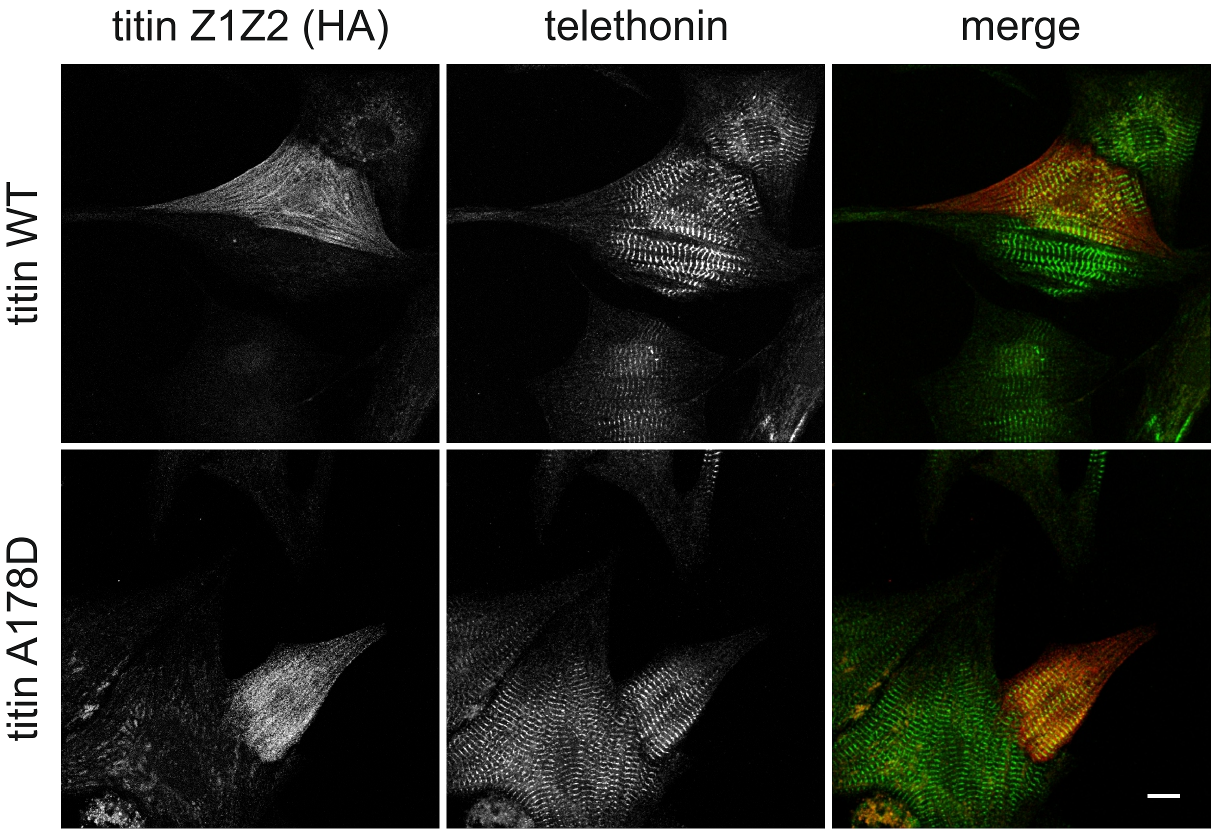

Figure S7

Table S1: Filtering criteria applied to all variants identified.

1. Within linkage region (LOD > 0), whereby both affected and unaffected individuals were considered in the linkage analysis

2. Exonic or splicing variant,
   i.e. with one of the following consequence terms: 'transcript_ablation', 'splice_donor_variant', 'splice_acceptor_variant', 'stop_gained', 'frameshift_variant', 'stop_lost', 'initiator_codon_variant', 'transcript_amplification', 'inframe_insertion', 'inframe_deletion', 'missense_variant', 'splice_region_variant'

3. Heterozygous and shared by both affected individuals (III-1 and III-4)

4. Called confidently by Platypus (flagged as 'PASS')

5. Overlapping neither segmental duplications nor repeats

6. Allele frequency in 1000 Genomes ≤ 1 % or not reported [1]

7. Observed no more than 7 times in WGS500 [2]

8. Observed no more than 50 times in ESP
   (Exome Variant Server, http://evs.gs.washington.edu/EVS/)

9. Observed no more than 50 times in UK10K
   (UK10K Project, http://www.uk10k.org)

10. Observed no more than 500 times in ExAC Browser
    (Exome Aggregation Consortium, http://exac.broadinstitute.org/)

Steps 1 to 10 are implemented in an automated script, further filtering steps are based on manual inspection:

11. Supporting evidence for the existence of affected transcript(s)

12. Evidence of expression of the gene in the heart both at RNA and protein level using multiple databases (see Expanded Materials)

13. Splice/intronic variants: considered if at crucial position (-2 to +2) or violating consensus rules at position -6 to -3 (for 5’ sites) or at position -3 for 3’ sites [3]

    Predicted to be tolerated using MaxEntScan
    (http://genes.mit.edu/burgelab/maxent/Xmaxentscan_scoreseq.html and http://genes.mit.edu/burgelab/maxent/Xmaxentscan_scoreseq_acc.html)

References Table S1

1. 1000 Genomes Project Consortium, Abecasis GR, Auton A, Brooks LD, DePristo MA, Durbin RM , et al. An integrated map of genetic variation from 1,092 human genomes. *Nature*. 2012;491:56-65.

2. Taylor JC, Martin HC, Lise S, Broxholme J, Cazier JB, Rimmer A , et al. Factors influencing success of clinical genome sequencing across a broad spectrum of disorders. *Nat Genet*. 2015;47:717-726.

3. Padgett RA. New connections between splicing and human disease. *Trends Genet*. 2012;28:147-154.

Table S2: Variants remaining after Platypus filtering (steps 1-10 of Table S1)

											Allele frequency				
**CHROMOSOME**	**POSITION**	**REFERENCE**	**ALTERATION**	**QUALITY**	**FILTER**	**GENE**	**CONSEQUENCE**	**AA_CHANGE**	**SIFT**	**POLYPHEN**	**1000G**	**UK10K(AC/AN)**	**ESP6500(AC/AN)**	**EXAC**	**Reason for exclusion**
16	66919133	G	A	1484	PASS	*PDP2*	missense_variant	E316K	deleterious(0)	probably_damaging(1)	0	1.3E-04	0	4.9E-05	n/a
2	179665172	G	T	1658	PASS	*TTN*	missense_variant	A178D	deleterious(0)	possibly damaging (0.734)	0	0	0	0	n/a
16	31495991	C	T	1852	PASS	*SLC5A2*	splice_region_variant & intron_variant				0	0	0	8.2E-06	expressed exclusively in kidney and testis; position -3 of a 3' splice junction, predicted to be tolerated
16	67208979	G	T	1242	PASS	*NOL3*	missense_variant	G45V		unknown(0)	0	0	0	2.5E-05	an artefact due to an incorrect, poorly supported transcript (ENST00000564860) present in Ensembl; synonymous R213R change in all other transcripts
2	176995495	C	T	1044	PASS	*HOXD8*	missense_variant	A134V	tolerated(0.08)	benign(0.31)	0	0	0	0	not present in affected individual III-6
2	179414205	A	G	1005	PASS	*TTN*	splice_region_variant & intron_variant				0	0	0	0	position -5 of a 3' splice junction, predicted to be tolerated (for detailed analysis see Table S3)

RNA and protein expression in the heart

**GENE**	**RNA**					**Protein**					**Comment**
	GeneCards	Expression Atlas (EMBL)	Protein Atlas	GeneHub	GTex	Protein Atlas	Human Protein Map	Proteomics DB	PaxDB	GeneCards	
TTN	✓	✓	✓	✓	✓	✓	✓	✓	✓	✓	*known cardiomyopathy gene*
PDP2	✓	✓	✓	✗	✓ (low)	✓	✗	✗	✗	✗	*low expression in the heart*

Table S3: Analysis and exclusion of an intronic/splice region variant in titin.

Position 2:179,414,205	Reference	Variant
base	A	G

Position - 5 position of 3' splice intron /exon 339 boundary (ENST00000589042)

Affected exon [1] Fibronectin domain in A-band titin

Percentage spliced in (PSI) of exon 339: 100 (DCM), 98 (controls)

**Percentage Spliced In (PSI) Prediction**		**Reference**
SPANR	difference in PSI score between refence and variant: 0.8 %	http://tools.genes.toronto.edu/

Conservation PhyloP poor

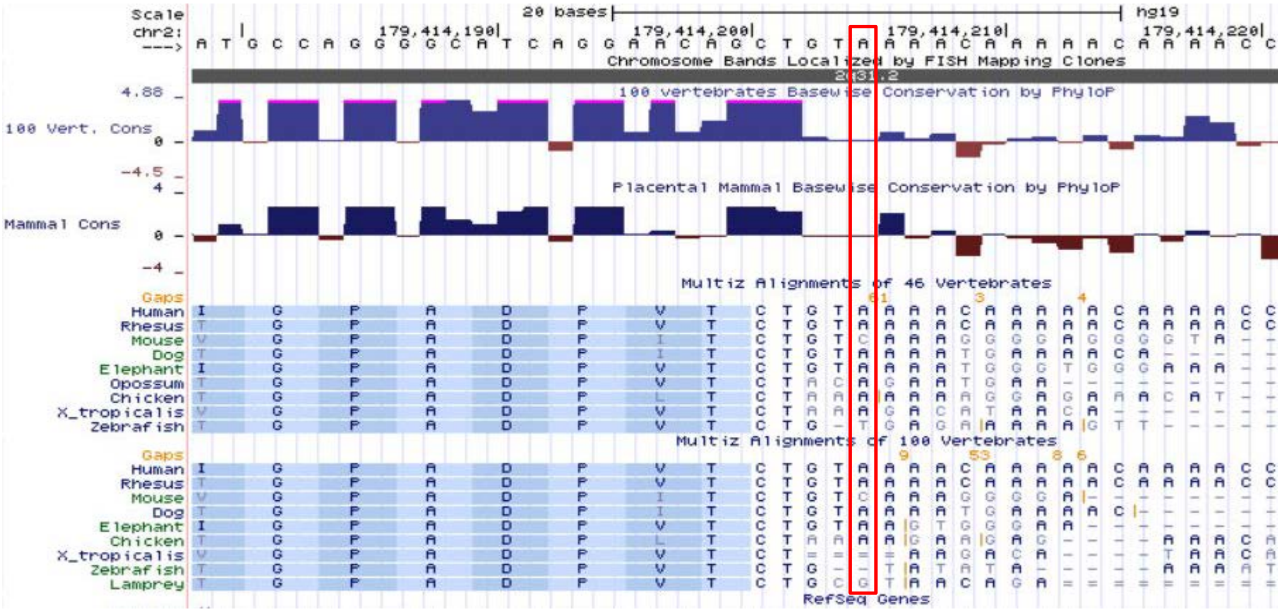

Splice Prediction

Tool	Reference	Variant	Difference	Percent	Consequence	Reference
MaxEntScan	11.08	9.94	1.14	10%	tolerated (< 15 %, [2])	http://genes.mit.edu/burgelab/maxent/Xmaxentscan_scoreseq_acc.html
VEP-MaxEntScan	11.08	10.90	0.15	1.4%	tolerated (< 15 %, [2])	
BDGP	0.99	0.98	0.01	1.0%	tolerated (cut off 0.40)	www.fruitfly.org/seq_tools/splice.html
Summary Alamut Visual v2.6	Predicted change at acceptor site 5 bps downstream: -7.8%				"not predictable"	
SpliceSiteFinder	93.8	92.4	1.4	1.5%		Alamut Visual (Interactive Biosoftware)
NNSPLICE	1.0	0.9	0.1	10.0%		Alamut Visual (Interactive Biosoftware)
GeneSplicer	7.5	4.7	2.8	37.0%		Alamut Visual (Interactive Biosoftware)
Human Splicing Finder	90.5	86.9	3.6	4.0%		Alamut Visual (Interactive Biosoftware)

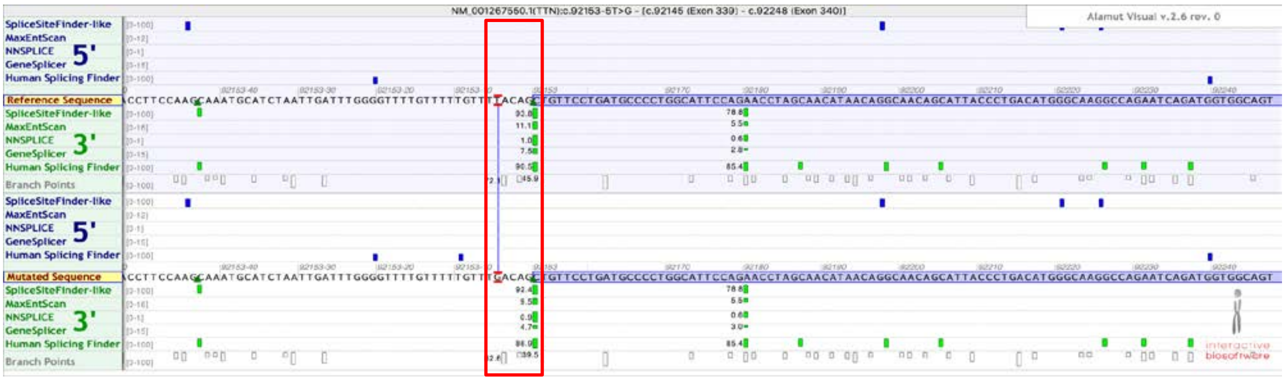

References

[1] Roberts AM, Ware JS, Herman DS, Schafer S, Baksi J, Bick AG , et al. Integrated allelic, transcriptional, and phenomic dissection of the cardiac effects of titin truncations in health and disease. *Science Translational Medicine* . 2015;7:270ra6.

[2] Houdayer C, Caux-Moncoutier V, Krieger S, Barrois M, Bonnet F, Bourdon V, et al. Guidelines for splicing analysis in molecular diagnosis derived from a set of 327 combined in silico/in vitro studies on BRCA1 and BRCA2 variants. *Hum Mutat.* 2012; 33(8):1228-38.

Table S4: Primer sequences

Sanger sequencing

TTN A178D

Forward 5’ –TCACCTGGTTTTGGAATTGG – 3’

Reverse 5’ –GGCCCCATTTAGACACAAAC – 3’

PDP2 E316K

Forward 5’ –CTGGAAGATGAGGTGACAAGG – 3’

Reverse 5’ –ATCTGAGGCCAGCACAAGG – 3’

Cloning primer (restriction sites in capital letters)

Telethonin amino acids 1-90 pEBG

Forward 5’ – tttGGATCCatggctacctcagagctgagc -3’

Reverse 5’ – tttGCGGCCGCttacggcagtacccgctggtag – 3’

Telethonin amino acids 1-167 pEBG

Forward 5’ – tttGGATCCatggctacctcagagctgagc -3’

Reverse 5’ – tttGCGGCCGCtcagcctctctgtgcttcctg -3’

Titin Z1Z2 pShuttle IRES hrGFP-2

Forward 5’- tttGCGGCCGCcccaccatgacaactcaagcaccgacg - 3’

Reverse 5’- tttCGATCGGaccttgaaccagtaattcagcag -3’

Titin Z1Z2 into pEGFP-N1

Forward 5’- tttCTCGAGatgacaactcaagcaccgacg – 3’

Reverse 5’- tttGGATCCcgaccttgaaccagtaattcagcag – 3’

Titin Z1Zr3 into pECFP-C1 (FRET)

Forward 5’ –tttctcgagccATGACAACTCAAGCACCGAC 3’

Reverse 5’ –ttggatcctcaGTAACTCAAGAAGCAATAAGA -3’

TelethoninΔC (amino acids 1-90) into pEYFP-C1 (FRET)

Forward 5’ –tttctcgagccATGGCTACCTCAGAGCTGAGC -3’

Reverse 5’ –ttggatcctcaCGGCAGTACCCGCTGGTAG – 3’

Mutagenesis *TTN* A178D

Forward 5’ – ACTCAGGGACCTATTCAGTAAATGaCACCAATAGCG – 3’

Reverse 5’ – CGCTATTGGTGtCATTTACTGAATAGGTCCCTGAGT – 3’
