# Supplementary material for: Combination of Whole Genome Sequencing, Linkage, and Functional Studies Implicates a Missense Mutation in Titin as a Cause of Autosomal Dominant Cardiomyopathy With Features of Left Ventricular Noncompaction
Source: Circ Cardiovasc Genet. 2016 Oct 18;9(5):426–35. doi: 10.1161/CIRCGENETICS.116.001431 (PMC5068189; doi:10.1161/CIRCGENETICS.116.001431)
Supplement: Supplementary file 2 [file hcg-9-426-s002.pdf]

DOI: 10.1161/CIRCGENETICS.116.001431

Combination of Whole Genome Sequencing, Linkage and Functional Studies Implicates a Missense Mutation in Titin as a Cause of Autosomal Dominant Cardiomyopathy with Features of Left Ventricular Non-Compaction

Running title: *Hastings et al.; Cardiomyopathy-causing missense mutation in TTN*

Robert Hastings, MBChB DPhil MRCP[1]*; Carin de Villiers, PhD[1]*; Charlotte Hooper, PhD[1]; Liz Ormondroyd, PhD, MSc[1]; Alistair Pagnamenta, PhD[2,3]; Stefano Lise, PhD[3]; Silvia Salatino, PhD[3]; Samantha JL Knight, PhD, CBiol, MSB, FRCPath[2,3]; Jenny C. Taylor, PhD[2,3]; Kate L. Thomson, BSc, FRCPath[1,4]; Linda Arnold, MSc[1]; Spyros D. Chatziefthimiou, PhD[5]; Petr V. Konarev, PhD[5, 6]; Matthias Wilmanns, PhD[5]; Elisabeth Ehler, PhD[7]; Andrea Ghisleni, MSc[7]; Mathias Gautel, MD, PhD[7]; Edward Blair, BMSc, MBChB[4]; Hugh Watkins, MD, PhD, FRCP[1]; Katja Gehmlich, PhD[1]

[1]Division of Cardiovascular Medicine in the Radcliffe Department of Medicine, University of Oxford; BHF Centre of Research Excellence; [2]NIHR Biomedical Research Centre Oxford & [3]Wellcome Trust Centre for Human Genetics, University of Oxford; [4]Department of Clinical Genetics, Churchill Hospital, Oxford University NHS Trust, Oxford, United Kingdom; [5]European Molecular Biology Laboratory, Hamburg, Germany; [6]Laboratory of Reflectometry and Small-Angle Scattering, A.V.Shubnikov Institute of Crystallography, Russian Academy of Sciences, Moscow, Russian Federation; [7]Randall Division of Cell and Molecular Biophysics and Cardiovascular Division, King's College London BHF Centre of Research Excellence, London, United Kingdom
*contributed equally

American Heart Association
Circulation: Cardiovascular Genetics

Correspondence:
Katja Gehmlich
Division of Cardiovascular Medicine
Radcliffe Department of Medicine, University of Oxford
Level 6, West Wing John Radcliffe Hospital, Headley Way
Oxford OX3 9DU, United Kingdom
Tel: ++44 1865 234902
Fax: ++44 1865 234681
E-mail: katja.gehmlich@cardiov.ox.ac.uk

Journal Subject Terms: Genetics; Cardiomyopathy; Basic Science Research

Abstract:

Background - High throughput next generation sequencing techniques have made whole genome sequencing accessible in clinical practice, however, the abundance of variation in the human genomes makes the identification of a disease-causing mutation on a background of benign rare variants challenging.

Methods and Results - Here we combine whole genome sequencing with linkage analysis in a three-generation family affected by cardiomyopathy with features of autosomal dominant left-ventricular non-compaction cardiomyopathy. A missense mutation in the giant protein titin is the only plausible disease-causing variant that segregates with disease amongst the eight surviving affected individuals, with interrogation of the entire genome excluding other potential causes. This A178D missense mutation, affecting a conserved residue in the second immunoglobulin-like domain of titin, was introduced in a bacterially expressed recombinant protein fragment and biophysically characterised in comparison to its wild-type counterpart. Multiple experiments, including size exclusion chromatography, small angle X-ray scattering and circular dichroism spectroscopy suggest partial unfolding and domain destabilisation in the presence of the mutation. Moreover, binding experiments in mammalian cells show that the mutation markedly impairs binding to the titin ligand telethonin.

Conclusions - Here we present genetic and functional evidence implicating the novel A178D missense mutation in titin as the cause of a highly penetrant familial cardiomyopathy with features of left-ventricular non-compaction. This expands the spectrum of titin's roles in cardiomyopathies. It furthermore highlights that rare titin missense variants, currently often ignored or left un-interpreted, should be considered to be relevant for cardiomyopathies and can be identified by the approach presented here.

American Heart Association.
Circulation
Cardiovascular Genetics

Key words: left ventricular noncompaction; cardiomyopathy; whole genome sequencing; titin; telethonin; missense mutation

Introduction

Cardiomyopathies (CM) are a diverse group of diseases affecting the heart muscle [1]; many of them are inherited and transmitted in autosomal dominant patterns. The first cardiomyopathy genes were identified by genome-wide linkage analysis in large families [2]. In practice, however, the small size of most families, or even the availability of members of larger families, often limits the power of linkage analysis. Recently, high throughput next generation sequencing (NGS) techniques have become widely accessible, making whole genome sequencing (WGS) cost- and time-effective. However, the abundance of variation in the human genome [3] makes it difficult to distinguish rare benign variants from rare disease-causing mutations in an isolated individual, even with growing knowledge of variants in population cohorts (e.g. >60,000 sequenced exomes in the ExAC database, http://exac.broadinstitute.org/). NGS poses, therefore, a significant clinical challenge: the capability to assess variants as pathogenic lags significantly behind variant identification, especially for non-synonymous point mutations [4, 5]. Algorithmic predictors are currently unable to accurately assess their exact impact on protein-protein interactions or even on protein folding. Experimental validation of genetic variants is therefore an increasingly indispensable component of NGS discoveries.

In the current study, we combine WGS with linkage analysis in a medium-sized family affected by cardiomyopathy with features of left-ventricular non-compaction cardiomyopathy (LVNC). By performing WGS in two family members, filtering against variants seen in normal population cohorts and using linkage information derived from single nucleotide polymorphism (SNP) arrays of 13 family members, we could identify a missense variant in the titin gene (*TTN*) as the most plausible cause of disease in the family. Functional data, generated from biophysical and protein binding experiments on this titin missense variant provide further support of a

American Heart Association. Circulation Cardiovascular Genetics

causative role in cardiomyopathy through domain misfolding and destabilisation, resulting in impaired binding to the ligand telethonin (also known as t-cap).

Methods

Clinical Evaluation

The study was approved by the Oxfordshire Research Ethics Committee B (REC Ref 09/H0605/3) and all subjects gave informed consent. A three-generational family with history of cardiomyopathy was recruited. Clinical assessment and genetic studies were performed in available family members, who had clinical examination, ECG, echocardiography (with contrast agent where appropriate) and cardiac MRI if possible. Diagnosis of cardiomyopathy was based on established criteria. The diagnosis of LVNC was based on published criteria from echocardiographic and/or cardiac MR imaging [6, 7]: the compaction ratio (CR), i.e. the ratio of the thickness of non-compacted to compacted myocardium >2.3 measured on MRI in diastole, or >2.0 on echocardiography in systole was used to diagnose LVNC.

Circulation Cardiovascular Genetics

Genetic Studies

SNP array genotyping was performed using the Illumina HumanCytoSNP-12v1 BeadChip (Illumina, San Diego, CA), containing nearly 300,000 genetic markers, according to the manufacturer's protocols. A refined subset of roughly 24,000 SNPs in approximate linkage equilibrium was generated using the software PLINK v1.07 [8] and the HapMap genotype file available from the PLINK website (http://pngu.mgh.harvard.edu/purcell/plink/). Linkage analysis of the SNP subset was performed using MERLIN v1.1.2 [9], specifying an autosomal dominant disease model. Genomic intervals with LOD scores > 0, compatible with segregation of variants in these regions, were selected for downstream analyses.

WGS was performed on genomic DNA extracted from peripheral blood as part of the WGS500 project as described previously [10].

Sequence reads from the affected individuals were mapped to the human reference genome (hs37d5 version of build 37) using STAMPY [11]. Duplicate reads were removed with PICARD (http://broadinstitute.github.io/picard/). The software Platypus (version 0.8.1, default parameters) [12] was used jointly on the two .bam files in order to call SNPs and short (< 50 bp) indels across both samples.

All the 5,946,161 identified variants were annotated with an in-house pipeline based on the Variant Effect Predictor (VEP) Ensembl framework (version 77) [13]. A number of additional databases were used to integrate the information provided by VEP (Table S1). Known associations with diseases were screened using HGMD (http://www.hgmd.cf.ac.uk/ac/index.php) and ClinVar [14].

Variants were filtered by in-house Python scripts based on criteria outlined in Table S1 (steps 1-10), followed by manual inspection (steps 11-13). The variants remaining after step 10 are documented in Results and in Tables S2, S3. Confirmatory Sanger sequencing was performed with the primers listed in Table S4.

Both SNP and WGS data were interrogated also for clinically relevant copy number variants (CNVs) using Nexus Copy Number 7.5.2 Discovery Edition (BioDiscovery, Hawthorne, CA; see Supplementary methods).

Functional characterisation of the titin missense variant

The mutation was introduced into human titin Z1Z2 constructs (amino acids 1-196, accession no. ACN81321.1) for bacterial and mammalian expression using Quikchange II XL (Agilent) with primers given in Table S4. Bacterial expression and purification was performed as previously

American Heart Association
Circulation
Cardiovascular Genetics

described [15]. Size exclusion chromatography - Tridetector analysis (light scattering, refractive index, and UV absorbance), small angle X-ray scattering (SAXS) experiments, circular dichroism spectroscopy, and thermolysin digests were essentially performed as described [15-18] and experimental details are given in Suppl. Material.

NRC cultures were established and transfected [16] using hemagglutinin(HA)-tagged expression constructs and counter-stained for titin T12 epitope [19] or telethonin (mouse monoclonal antibody, Santa Cruz) 48 hrs post transfection and analysed by confocal microscopy.

GST pulldown assays were performed as described [20] using mammalian expression constructs for telethonin amino acids 1-90 and 1-167 fused to GST, and titin Z1Z2 fused to GFP (pEGFP-N1, Clontech) in transfected COS-1 cells. Förster Resonance Energy Transfer (FRET) experiments from transfected COS-1 cells and the assessment of reduced protein stability in NRC and COS-1 cells are described in the Suppl. Material.

Circulation Cardiovascular Genetics

Results

The proband was a 20yr old male (II-3 in Figure 1A) who died suddenly in hospital in 1970 having presented with rapidly decompensating congestive heart failure; at post mortem his heart (680 g) had evidence of dilatation and both macroscopic and microscopic hypertrophy but no myocyte disarray. His brother (II-4) was later found to have an enlarged heart with wall thickness at the upper limit of normal and marked hypertrabeculation. The proband’s sister (II-2) presented with a non ST-elevation myocardial infarct due to coronary embolus at the age of 61. LVNC with mild LV dilatation and apical hypertrophy was diagnosed at this time (Figure 1B, C). Cascade screening identified the same condition in further family members with consistent clinical features of adult onset cardiomyopathy with features of LVNC. Five affected family members had sufficient non-compaction to meet the diagnostic criteria for LVNC while three

others with early or mild disease had lesser extent of hypertrabeculation but clear evidence of cardiomyopathy with LV dilatation and/or systolic dysfunction (Figures 1A, S1, Table 1). Aside from the proband who had advanced congestive failure, there were no arrhythmic features in any affected family member, nor were there any extra-cardiac (e.g. neuromuscular) manifestations.

Identification of TTN mutation A178D segregating with disease

Affected first cousins III-1 and III-4 were selected for WGS. Sequencing was performed by Illumina Cambridge as 100bp paired-end reads to a mean coverage of 56.9x and 52.0x respectively, such that 99 % of the genome was covered at 20x or more in both samples, identifying 5,946,161 variants shared by the two individuals. In addition, SNP arrays were performed on all individuals of the family (except II-3 and III-2, Figure 1A). Neither the SNP array nor WGS data revealed likely causative CNVs.

American Heart Association. Circulation Cardiovascular Genetics

Genomic regions identical by descent were identified through linkage analysis (see Methods, Figure S2) and out of the 100,789 candidate variants within the three linkage regions (on chromosomes 2, 9 and 16), potentially pathogenic ones were selected based on an autosomal dominant model, caused by a rare heterozygous mutation. Variants were filtered accordingly by in-house Python scripts and the remaining six variants were manually inspected (Table S2). Four of them were excluded: one is assumed to be an artefact due to an incorrect transcript being present in Ensembl and another variant did not segregate with disease in the family; two splice variants were predicted to be silent (at positions -5 and -3 of a 3' splice junction, respectively, for details see Table S3). Only two final candidate variants were considered conceivably linked to the phenotype: missense changes in *PDP2* and *TTN,* respectively (Table S2). *PDP2* codes for pyruvate dehyrogenase phosphatase catalytic subunit 2 and has low expression levels in the heart. Although the change E316K is predicted to be damaging by Polyphen and SIFT

algorithms (Table S2), a heterozygous loss-of-function in this enzyme would not be expected to produce a phenotype, and indeed heterozygous loss-of-function mutations in *PDP1* are clinically silent [21]. The variant is not plausible as a cause of a penetrant dominant disorder because it is found six times in 121,412 alleles in the ExAC database. Six instances would equal at least 10 % of all expected LVNC cases in ExAC, assuming a maximal prevalence of 1:1,000 for the disease [22]. This appears an implausibly high percentage for a novel, unpublished disease-causing variant. In support, in the two largest clinical cardiomyopathy cohorts published to date, the most common reported pathogenic variant (*MYBPC3*, p.Arg502Trp) detected in 104 out of 6179 HCM cases (1.7%, 95CI 1.4-2.0%), was only observed 3 times in ExAC (3/120,674) with all other pathogenic variants for HCM or DCM being present 0 or 1 times only [23].

American Heart Association
Circulation
Cardiovascular Genetics

The second variant is found in *TTN*, the gene which codes for titin, an abundant skeletal muscle and heart specific protein with crucial functions [24, 25] (and reviewed in [26]). Mutations in titin have been associated with CM and skeletal myopathy (reviewed in [27]). The identified missense variant c.533C>A in *TTN*, which codes for a p.A178D change at the amino-acid level, is absent in ExAC. Sanger sequencing confirmed the co-segregation of the heterozygous mutation with disease in all affected individuals of the family (Figures 1, S3A, LOD score 2.1). Thus comprehensive whole genome analysis reveals this as the most plausible causative mutation in the family.

Functional studies

Prediction of deleterious effects of the mutation

Each single molecule of the giant protein titin spans half a sarcomere from the Z-disk to the M-band [28]. The first two Immunoglobulin-like (Ig) domains (Z1Z2) of titin are located in the Z-disk and form a super-stable complex with telethonin [29]. The A178 position is evolutionarily very

well conserved back to zebrafish and lamprey. Additionally, A178 is located in a highly conserved structural section (Figure S3B), the β-strand F of the second Ig-domain of Z1Z2, neighbouring the β-strand G of titin Z2 which forms a strong and extended interaction with the β-strands of telethonin ([30], Figure 2A). The A178D mutation is predicted to directly affect the β-strands B and C as well as the loop connecting the β-strands B and C due to steric hindrance of D178 with V127 and P133 respectively (Figure 2B). Thus, the insertion of a charged residue in this position is likely to have significant impact on the secondary structure of this domain and could potentially cause misfolding of the protein.

Altered protein characteristics of purified titin Z1Z2 A178D recombinant fragment

To assess how the A178D mutation affects the folding and stability of the protein, recombinant titin Z1Z2 WT and A178D were expressed in *E. coli* and purified under native conditions. Of note, the yield of the soluble protein fraction was consistently lower for A178D compared to WT preparations, despite equal total expression levels (data not shown). Circular dichroism (CD) spectroscopy demonstrated a typical β-sheet signature for WT Z1Z2 (Figure 3A). In contrast, the spectrum for Z1Z2 A178D differs significantly: Although the characteristic negative band at 216 nm is still present, but slightly shifted, there was no significant positive band at around 200 nm. The absence of this band, associated with β-sheet conformation, and the presence of a negative peak at around 198 nm, characteristic of random coil structures, indicate that the Z1Z2 A178D mutant is partially unfolded.

In support, thermal denaturation experiments for Z1Z2 A178D showed high fluorescence signal already at low temperatures, suggesting solvent exposed hydrophobic residues due to partial unfolding. No melting temperature can be deducted for titin Z1Z2 A178D, in contrast to the WT protein, which has a melting temperature of 62 ºC, typical for Ig domains (Figure S4).

American Heart Association. Circulation Cardiovascular Genetics

DOI: 10.1161/CIRCGENETICS.116.001431

SAXS experiments confirmed the presence of unfolded parts/flexible domains in Z1Z2 A178D, as shown by the Kratky plot (Figure S5A), whereas Z1Z2 WT displays a typical profile for folded structures.

The domain destabilisation as a consequence of partial unfolding is evidenced by the formation of higher oligomers (approx. 20-mers) for the Z1Z2 A178D mutant *in vitro*: Size exclusion chromatography and Tridetector analysis revealed that in contrast to the monomeric Z1Z2 WT, the A178D mutant eluted in two peaks, corresponding predominantly to higher molecular aggregates and to a lesser extent to dimeric protein (Figure 3B, Table 2). SAXS measurements also confirmed that Z1Z2 WT is monomeric, whereas Z1Z2 A178D is found in a higher oligomeric state (Figure S5B, Table 2).

In conclusion, the mutation A178D leads to partial misfolding of bacterially expressed Z1Z2 protein fragment.

Reduced stability of titin Z1Z2 A178D as a consequence of the partial misfolding

When performing denaturing gel electrophoresis, a degradation product was observed exclusively for Z1Z2 A178D preparations (arrowhead in Figure 4A) and upon thermolysin treatment, only Z1Z2 A178D showed rapid degradation, while Z1Z2 WT was resistant to the protease treatment (Figure 4B). In addition, Z1Z2 A178D showed reduced stability when expressed in neonatal rat cardiomyocytes and COS-1 cells (Figures 4C, S6), suggesting that the mutation destabilises Z1Z2 also in a physiological, cellular environment. However, formation of large aggregates was not observed in transfected cells expressing Z1Z2 A178D (Figures 4D, S7).

Impaired binding to telethonin

Localisation of transfected Z1Z2 was not altered in the presence of the A178D mutation (Figure 4D). To assess the consequences of the mutation on binding telethonin, semi-quantitative GST-

American Heart Association
Circulation Cardiovascular Genetics

pulldown assays were performed with titin Z1Z2 and telethonin co-expressed in mammalian cells. Z1Z2 A178D showed impaired binding to two telethonin constructs (Figure 5A, B). The interaction between titin and telethonin was further quantified in FRET experiments, where close proximity of proteins in a complex allows energy transfer from Cyan Florescent Protein (CFP) to Yellow Fluorescent Protein (YFP) between two fusion protein constructs [31]. By introducing the A178D mutation into a Z1Zr3-CFP construct, FRET efficiency to telethonin-YFP was almost abolished (Figure 5C, D), validating and quantifying the observation that A178D impairs binding to telethonin in the cellular context.

Taken together, our functional data suggest that the A178D mutant may affect protein folding, stability and impairs binding to telethonin, thus supporting its pathogenic potential.

American Heart Association

Circulation Cardiovascular Genetics

Discussion

In this study, we present a three-generation family with multiple individuals affected by cardiomyopathy with features of LVNC, systolic impairment and an autosomal dominant inheritance pattern. Of note, the affected family members show a consistent phenotype with prominent hypertrabeculation as the main abnormality in the majority; this is relatively unusual as it is more typical to see LVNC in individual members of families with other forms of cardiomyopathy.

We employed a combination of WGS in two affected individuals and linkage analysis in 13 family members; this approach identified only two rare candidate variants across the whole genome that segregated with the autosomal dominant cardiomyopathy. Since one of the identified genes (*PDP2*) is barely expressed in the heart, and the variant appears in implausible high numbers in the ExAC database, it is extremely unlikely to be disease-causative. In contrast, titin, the gene affected by the other missense variant (*TTN* p.A178D), has crucial functions in the

heart and is a known disease gene for cardiomyopathies (see below). Despite the fact that the family is too small for traditional genome-wide linkage analysis to identify the genetic cause of the disease (the LOD score of 2.1, i.e. odds ratio 1:125, is well below the threshold of 3.0, i.e. odds ratio 1:1000), interrogation of the entire genome adds substantial weight to a likely causative role of the titin missense mutation for disease: no other plausible mutations, including larger genomic re-organisations (CNVs), were detected in any other genes in the linkage regions and the remainder of the genome is excluded by negative LOD scores.

Titin has been implicated in cardiac and skeletal muscle disease, occasionally involving a combination of both. Mutations in this gene have been described in various forms of CM, such as Dilated CM, Arrhythmogenic Right Ventricular CM, Hypertrophic CM and Restrictive CM (reviewed in [27]). Truncating variants in titin (TTNtv) are the most frequent genetic finding in idiopathic Dilated CM, being present in 15-25 % of the cases [32] and are also frequent in Peripartum CM (15 %) [33]. However, penetrance appears to be low, as TTNtv are also found in approx. 1 % of normal populations and hence the large majority of carriers do not manifest with disease [34]. More recent work [35] showed that Dilated CM causing TTNtv are enriched in the sarcomeric A-band region, whereas TTNtv found in control cohorts tend to spare the A-band region and are in exons with low usage in cardiac transcripts. An internal promotor in titin rescuing TTNtv N-terminally of the A-band region may explain this phenomenon [36].

Titin missense mutations have been identified in Dilated and Hypertrophic CM cohorts [4, 37, 38]. A causative role for *TTN* p. W976R in Dilated CM is well supported by co-segregation within a large family and functional data [39, 40]. However, generally, titin missense mutations are challenging to interpret, as rare benign variants are common in normal population cohorts. In the ExAC database, more than a third of the individuals carry a rare missense variant in titin (21,939

American Heart Association. Circulation Cardiovascular Genetics

missense variants with <0.01 % allelic frequency in 58,687 exomes), and although a proportion of these may represent recessive pathogenic alleles [27], only a very small fraction will be disease-causing with dominant inheritance. Hence, clinical practitioners require co-segregation information to assign causality as bioinformatic prediction tools can only give probabilistic data [4, 37]. As we document here, interrogation of the entire genome combined with linkage analysis can help to narrow down lists of potential causative variants, even in small families.

Our finding of *TTN* p.A178D in a family with features of LVNC expands the spectrum of titinopathies: to our knowledge, this is the first report of a titin missense mutation implicated in cardiomyopathy with predominant features of LVNC and one of the first titin missense mutations supported by robust genome-wide genetics and detailed functional data. The latter suggests a likely pathogenic role of titin A178D by a) evidence of protein degradation, partial unfolding and domain destabilisation *in vitro*, b) protein destabilisation in two cellular systems and c) altered binding properties to the ligand telethonin. Although extrapolations from such *in vitro* experiments on isolated domains to the full length giant protein are not without uncertainty, such parameters will be useful complements in the future studies of other *TTN* missense variants. It is currently unclear how this particular mutation leads to this distinct phenotype, and more insight into the biology of Z-disk titin is needed to understand the underlying disease pathways. This will be addressed with the help of model organisms [36, 41] or patient-derived induced pluripotent stem cell derived cardiomyocytes [40], focussing on the titin-telethonin complex [29] and its downstream signalling targets [42] in future work.

American Heart Association. Circulation Cardiovascular Genetics

Acknowledgments” We thank Stephan Lange (UCSD) for a titin Z1Z2 expression constructs. SAXS data were collected at the beamline P12, operated by EMBL, Hamburg unit, at the PETRA III storage ring (DESY, Hamburg, Germany). We gratefully thank Dmitry Svergun and

his group for help with the SAXS data, the SPC facility at EMBL Hamburg for technical support and Annabel Parret for her help with the Tridetector Analysis.

Sources of Funding: KG is supported by British Heart Foundation Grants (FS/12/40/29712, PG/15/113/31944). KG, RH and HW acknowledge support from the BHF Centre of Research Excellence, Oxford (grant codes HSRNWBY, HSRNWB11 and RE/13/1/30181). KLT is the recipient of a National Institute for Health Research (NIHR) doctoral fellowship (NIHR-HCS-D13-04-006). This publication includes independent research supported also by the NIHR Biomedical Research Centre, Oxford. The work was supported also by funding from the Wellcome Trust Core Award Grant Number 090532/Z/09/Z. The views expressed are those of the authors and not necessarily those of the Department of Health or Wellcome Trust. MG and AG were supported by the EU MUZIC network, the MRC and the Leducq Foundation. MG holds the BHF Chair of Molecular Cardiology.

Disclosures: None.

American Heart Association

Circulation
Cardiovascular Genetics

References:

1. Watkins H, Ashrafian H, Redwood C. Inherited cardiomyopathies. *N Engl J Med.* 2011;364:1643-1656.

2. Jarcho JA, McKenna W, Pare JA, Solomon SD, Holcombe RF, Dickie S, et al. Mapping a gene for familial hypertrophic cardiomyopathy to chromosome 14q1. *N Engl J Med*. 1989;321:1372-1378.

3. 1000 Genomes Project Consortium, Abecasis GR, Auton A, Brooks LD, DePristo MA, Durbin RM , et al. An integrated map of genetic variation from 1,092 human genomes. *Nature.* 2012;491:56-65.

4. Haas J, Frese KS, Peil B, Kloos W, Keller A, Nietsch R , et al. Atlas of the clinical genetics of human dilated cardiomyopathy. *Eur Heart J.* 2015;36:1123-1135a.

5. Watkins H. Assigning a causal role to genetic variants in hypertrophic cardiomyopathy. *Circ Cardiovasc Genet.* 2013;6:2-4.

6. Jenni R, Oechslin E, Schneider J, Attenhofer Jost C, Kaufmann PA. Echocardiographic and pathoanatomical characteristics of isolated left ventricular non-compaction: a step towards classification as a distinct cardiomyopathy. *Heart.* 2001;86:666-671.

7. Petersen SE, Selvanayagam JB, Wiesmann F, Robson MD, Francis JM, Anderson RH , et al. Left ventricular non-compaction: insights from cardiovascular magnetic resonance imaging. *J Am Coll Cardiol*. 2005;46:101-105.

8. Purcell S, Neale B, Todd-Brown K, Thomas L, Ferreira MA, Bender D , et al. PLINK: a tool set for whole-genome association and population-based linkage analyses. *Am J Hum Genet*. 2007;81:559-575.

9. Abecasis GR, Cherny SS, Cookson WO, Cardon LR. Merlin--rapid analysis of dense genetic maps using sparse gene flow trees. *Nat Genet*. 2002;30:97-101.

10. Taylor JC, Martin HC, Lise S, Broxholme J, Cazier JB, Rimmer A , et al. Factors influencing success of clinical genome sequencing across a broad spectrum of disorders. *Nat Genet*. 2015;47:717-726.

11. Lunter G, Goodson M. Stampy: a statistical algorithm for sensitive and fast mapping of Illumina sequence reads. *Genome Res*. 2011;21:936-939.

12. Rimmer A, Phan H, Mathieson I, Iqbal Z, Twigg SR, Consortium WGS , et al. Integrating mapping-, assembly- and haplotype-based approaches for calling variants in clinical sequencing applications. *Nat Genet*. 2014;46:912-918.

13. McLaren W, Pritchard B, Rios D, Chen Y, Flicek P, Cunningham F. Deriving the consequences of genomic variants with the Ensembl API and SNP Effect Predictor. *Bioinformatics*. 2010;26:2069-2070.

14. Landrum MJ, Lee JM, Riley GR, Jang W, Rubinstein WS, Church DM , et al. ClinVar: public archive of relationships among sequence variation and human phenotype. *Nucleic Acids Res*. 2014;42:D980-985.

15. Zou P, Gautel M, Geerlof A, Wilmanns M, Koch MH, Svergun DI. Solution scattering suggests cross-linking function of telethonin in the complex with titin. *J Biol Chem*. 2003;278:2636-2644.

16. Geier C, Gehmlich K, Ehler E, Hassfeld S, Perrot A, Hayess K , et al. Beyond the sarcomere: CSRP3 mutations cause hypertrophic cardiomyopathy. *Hum Mol Genet*. 2008;17:2753-2765.

17. Reinhard L, Mayerhofer H, Geerlof A, Mueller-Dieckmann J, Weiss MS. Optimization of protein buffer cocktails using Thermofluor. *Acta Crystallogr Sect F Struct Biol Cryst Commun*. 2013;69:209-214.

18. Shaya D, Kreir M, Robbins RA, Wong S, Hammon J, Bruggemann A , et al. Voltage-gated sodium channel (NaV) protein dissection creates a set of functional pore-only proteins. Proc Natl Acad Sci U S A. 2011;108:12313-12318.

American Heart Association
Circulation Cardiovascular Genetics

19. Furst DO, Osborn M, Nave R, Weber K. The organization of titin filaments in the half-sarcomere revealed by monoclonal antibodies in immunoelectron microscopy: a map of ten nonrepetitive epitopes starting at the Z line extends close to the M line. *J Cell Biol*. 1988;106:1563-1572.

20. Gehmlich K, Asimaki A, Cahill TJ, Ehler E, Syrris P, Zachara E , et al. Novel missense mutations in exon 15 of desmoglein-2: role of the intracellular cadherin segment in arrhythmogenic right ventricular cardiomyopathy? *Heart Rhythm*. 2010;7:1446-1453.

21. Cameron JM, Maj M, Levandovskiy V, Barnett CP, Blaser S, Mackay N , et al. Pyruvate dehydrogenase phosphatase 1 (PDP1) null mutation produces a lethal infantile phenotype. *Hum Genet*. 2009;125:319-326.

22. Carrilho-Ferreira P, Almeida AG, Pinto FJ. Non-compaction cardiomyopathy: prevalence, prognosis, pathoetiology, genetics, and risk of cardioembolism. *Curr Heart Fail Rep*. 2014;11:393-403.

23. Walsh R, Thomson KL, Ware JS, Funke BH, Woodley J, McGuire KJ , et al. Reassessment Of Mendelian Gene Pathogenicity Using 7,855 Cardiomyopathy Cases And 60,706 Reference Samples. *Genet Med*. 2016 Aug 17. doi: 10.1038/gim.2016.90. [Epub ahead of print]

24. Labeit S, Kolmerer B, Linke WA. The giant protein titin. Emerging roles in physiology and pathophysiology. *Circ Res*. 1997;80:290-294.

25. Wang K, McClure J, Tu A. Titin: major myofibrillar components of striated muscle. *Proc Natl Acad Sci U S A*. 1979;76:3698-3702.

26. Gerull B. The Rapidly Evolving Role of Titin in Cardiac Physiology and Cardiomyopathy. *Can J Cardiol*. 2015;31:1351-1359.

27. Chauveau C, Rowell J, Ferreiro A. A rising titan: TTN review and mutation update. *Hum Mutat*. 2014;35:1046-1059.

28. Hidalgo C, Granzier H. Tuning the molecular giant titin through phosphorylation: role in health and disease. *Trends Cardiovasc Med*. 2013;23:165-171.

29. Bertz M, Wilmanns M, Rief M. The titin-telethonin complex is a directed, superstable molecular bond in the muscle Z-disk. *Proc Natl Acad Sci U S A*. 2009;106:13307-133310.

30. Zou P, Pinotsis N, Lange S, Song YH, Popov A, Mavridis I , et al. Palindromic assembly of the giant muscle protein titin in the sarcomeric Z-disk. *Nature*. 2006;439:229-233.

31. Truong K, Ikura M. The use of FRET imaging microscopy to detect protein-protein interactions and protein conformational changes in vivo. *Curr Opin Struct Biol*. 2001;11:573-578.

32. Herman DS, Lam L, Taylor MR, Wang L, Teekakirikul P, Christodoulou D , et al. Truncations of titin causing dilated cardiomyopathy. *N Engl J Med*. 2012;366:619-628.

33. Ware JS, Li J, Mazaika E, Yasso CM, DeSouza T, Cappola TP , et al. Shared Genetic Predisposition in Peripartum and Dilated Cardiomyopathies. *N Engl J Med*. 2016;374:233-241.

34. Watkins H. Tackling the achilles' heel of genetic testing. *Sci Transl Med*. 2015;7:270fs1.

35. Roberts AM, Ware JS, Herman DS, Schafer S, Baksi J, Bick AG , et al. Integrated allelic, transcriptional, and phenomic dissection of the cardiac effects of titin truncations in health and disease. *Sci Transl Med*. 2015;7:270ra6.

36. Zou J, Tran D, Baalbaki M, Tang LF, Poon A, Pelonero A , et al. An internal promoter underlies the difference in disease severity between N- and C-terminal truncation mutations of Titin in zebrafish. *Elife*. 2015;4:e09406.

37. Begay RL, Graw S, Sinagra G, Merlo M, Slavov D, Gowan K , et al. Role of Titin Missense Variants in Dilated Cardiomyopathy. *J Am Heart Assoc*. 2015;4. pii: e002645.

38. Lopes LR, Zekavati A, Syrris P, Hubank M, Giambartolomei C, Dalageorgou C , et al. Genetic complexity in hypertrophic cardiomyopathy revealed by high-throughput sequencing. *J Med Genet*. 2013;50:228-239.

39. Gerull B, Gramlich M, Atherton J, McNabb M, Trombitas K, Sasse-Klaassen S , et al. Mutations of TTN, encoding the giant muscle filament titin, cause familial dilated cardiomyopathy. *Nat Genet.* 2002;30:201-204.

40. Hinson JT, Chopra A, Nafissi N, Polacheck WJ, Benson CC, Swist S , et al. HEART DISEASE. Titin mutations in iPS cells define sarcomere insufficiency as a cause of dilated cardiomyopathy. *Science*. 2015;349:982-986.

41. Gramlich M, Pane LS, Zhou Q, Chen Z, Murgia M, Schotterl S , et al. Antisense-mediated exon skipping: a therapeutic strategy for titin-based dilated cardiomyopathy. *EMBO Mol Med*. 2015;7:562-576.

42. Knoll R, Linke WA, Zou P, Miocic S, Kostin S, Buyandelger B , et al. Telethonin deficiency is associated with maladaptation to biomechanical stress in the mammalian heart. *Circ Res*. 2011;109:758-769.

Table 1: Summary of clinical findings.

Symbol	Sex	Age*	Clinical status †	Genetic status	Trabeculation compaction ratio (CR) ‡	IVS D	PW D	LVED D	LVES D	EF	ECG	Comments
I-1	M	87	affected	TTN A178D	2.5 (echo)	24	13	40	35	42	left axis deviation, anteroseptal Q wave	Marked ASH with low EF, CVA, hypertension; meets diagnostic criteria for LVNC (echo).
I-2	F	72	unaffected	WT	1.8 (echo)	12	11	48	30	76	AF, paced	LVNC excluded (echo), structurally normal heart at age 70, moderate concentric LVH by age 85
II-2	F	61	affected	TTN A178D	2.0 (echo)	9	10	53	36	47	left axis deviation	Mildly thickened apical segments, cardiac embolus at 61y; meets diagnostic criteria for LVNC (echo).
II-3	M	20	assumed affected	no DNA								Rapidly progressive HF with sudden death at 20y (1970); hypertrophy and dilatation at post mortem.
II-4	M	37	affected	TTN A178D	<2 (MRI)	12	12	64	44	57	normal	Dilated LV, late Gd on MRI, hypertension.
II-6	M	42	unaffected	WT		11	9	50	30	78	normal	
III-1	M	27	affected	TTN A178D	2.5 (echo)	12	11	64	42	63	QRS 120 msec	Mild regional systolic dysfunction; meets diagnostic criteria for LVNC (echo).

III-2	M	21	unclassified	no DNA	0.8 (echo)	13	13	54	37	68	normal	Mild concentric LVH
III-3	M	32	affected	TTN A178D	2.8 (MRI)	11	13	52	35	68	normal	Hypertension; meets diagnostic criteria for LVNC (MRI)
III-4	F	23	affected	TTN A178D	2.6 (MRI)	8	9	46	32	58	normal	Meets diagnostic criteria for LVNC (MRI)
III-5	M	25	affected	TTN A178D	2.0-2.5 (MRI)	10	10	46	27	51	inferior T-wave inversion	Hypokinesia apical LV incl. septum; borderline for diagnostic criteria for LVNC (MRI)
III-6	M	23	affected	TTN A178D	1.5 (MRI)	8	9	53	34	48	Q wave & T-wave inversion in lead III	Mild DCM, faint late Gd, borderline dilated LV with mildly impaired function, inferior hypokinesia
III-7	M	21	unclassified	WT	1.6 (MRI)	9	7	55	38	59	normal	Documented myocarditis at 21y (MRI)

Circulation Cardiovascular Genetics
American Heart Association.

Cardiac dimensions (IVSD – Interventricular Septal Thickness at Diastole, PWD –Posterior Wall Thickness at Diastole, LVEDD – Left Ventricular End Diastolic Diameter, LVESD – Left Ventricular End Systolic Diameter) are given in mm Abbreviations: EF – Ejection Fraction (in %). ASH – Asymmetric Septal Hypertrophy, CVA – Cerebrovascular Accident, LVH – Left Ventricular Hypertrophy, Gd – Gadolinium, HF – Heart Failure, AF – Atrial Fibrillation, DCM – Dilated CM. Blank cells indicate no data available.

* Age of diagnosis or first clinical assessment, however parameters of most recent cardiac assessment are given (with exception of I-2 where data at first assessment aged 70 are given and II-7, where the last assessment before myocarditis is shown).

† Clinical status 'affected' means affected by cardiomyopathy. Whether individuals meet diagnostic criteria for LVNC is shown in the Comments column (in brackets shown whether MRI or echo criteria have been used).

‡ For the definition of trabeculation compaction ratio (CR) see Methods section, in brackets the mode of imaging is indicated. Representative MRI images are shown in Figure S1.

Table 2: Biophysical characterisation of recombinant Z1Z2 WT and A178D protein fragments

	Titin Z1Z2 WT	***Titin Z1Z2 A178D***
Calculated molecular weight (kDa)	22.7	22.8
**Size exclusion chromatography**		
Retention time (mL)	15.7	9.9 (1st peak)<br>14.4 (2nd peak)
**Static Light Scattering**		
Molecular weight (kDa)	21 ± 2	452 ± 45 (1st peak)<br>45 ± 4 (2nd peak)
**Small Angle X-ray Scattering**		
V_p excluded volume of the hydrated particle (nm^3)	40 ± 5	305 ± 20
R_g radius of gyration (nm)	3.10 ± 0.05	6.8 ± 0.1
D_{max} maximum particle size (nm)	10.5 ± 0.5	25.0 ± 1.0
Normalized Kratky plot	folded	partly unfolded/ flexible domains

Circulation Cardiovascular Genetics
American Heart Association.

Figure Legends:

Figure 1: A – Pedigree of the family, males depicted as squares, females as circles, slanted symbols deceased individuals. Clinically affected individuals are marked in grey, unaffected are shown in white, "?" means unclassified clinical status. The presence of the *TTN* p.A178D mutation is indicated ("+"present, "-"absent, ND not determined.) Individuals selected for WGS are marked with thicker symbols (III-1 and III-4). B – Echocardiogram images showing the characteristic 'spongy' appearance of non-compaction in individual II-2 with and without contrast. C – Echocardiogram image from individual II-4 showing significant dilatation, but maintaining a thickened myocardium and preserved ejection fraction.

American Heart Association.
Circulation
Cardiovascular Genetics

Figure 2: A – Position of the *TTN* p. A178D on a structural model (pdb: 1YA5) of the titin Z1Z2 domains (purple) in complex with telethonin (pink). B – Close-up of the site of mutation. The red discs show van der Waals overlaps or steric clashing that A178D is predicted to cause with valine127 and proline133. The figures of the crystal structure were generated by pymol (http://www.pymol.org).

Figure 3: A – CD spectroscopy of purified titin Z1Z2 fragments (WT solid line and A178D dashed line). B – Size exclusion chromatography for titin Z1Z2 fragments (WT solid line and A178D dashed line). Z1Z2 WT elutes as monomeric protein ($), whereas peaks corresponding to dimer (*) and higher molecular aggregates (#) are observed for Z1Z2 A178D.

Figure 4: Destabilisation of the titin Z1Z2 fragment in the presence of the A178D mutation: A – Denaturing gel-electrophoresis of purified titin Z1Z2 fragments (WT and A178D) expressed in *E. coli*. The WT fragment is detected as a single band of 23 kD (arrow). Only for Z1Z2 A178D a degradation product (arrowhead) is observed. The position of marker proteins and their size (in kD) is indicated. B – Titin Z1Z2 protein fragments (WT – left, A178D – right) were incubated with protease thermolysin for the length indicated. Control: titin Z1Z2 protein sample without thermolysin. A stable degradation fragment of approx. 15 kD is observed for the mutant titin Z1Z2. The position of marker proteins and their size (in kD) is indicated. C – Decreased stability of titin Z1Z2 A178D in NRC: NRC were infected in duplicates with adenoviral particles for HA-tagged titin Z1Z2 (WT or A178D, MOI 5). Infection with parental empty vector (GFP) and non-infected cells (NI) served as controls. Steady-state titin fragment protein amount was assayed by Western blotting for the HA-tag. Probing for hrGFP served as infection control and probing for endogenous GAPDH served as loading control. Despite equal infection rates (for confirmatory control experiments see Figure S6A), less titin A178D protein fragment was detected, indicating reduced stability in NRC. D – Localisation of titin Z1Z2 in NRC: Cells were transfected with constructs coding for HA-tagged titin Z1Z2 WT (top, first row) or titin Z1Z2 A178D (bottom, first row) mutant protein fragment and counterstained for endogenous titin with T12 antibody (middle row, Z-disk proximal epitope, but not recognising the transfected titin Z1Z2 protein fragment). Merged images are shown in the third row, HA shown in red, endogenous titin in green. Scale bar represents 10 microns.

Figure 5: Functional implications of the titin Z1Z2 A178D mutation. A – Semi-quantitative GST-pulldown assays using telethonin fragments fused to GST (left aa 1-90, right full length)

American Heart Association. Circulation Cardiovascular Genetics

and titin Z1Z2 fragments (WT and A178D as indicated) as GFP fusions expressed in COS-1 cells. Bound titin-GFP fragments are detected by Western blotting (top row), input lysate controls are shown in the second row. Pulled down GST-telethonin fragments are shown in row three, as well as lysate controls (bottom row). B – Quantification of GST-pulldown experiments from panel A visualises reduced binding of titin Z1Z2 to telethonin in the presence of the A178D mutation, values are expressed as bound protein relative to lysate, with the first WT experiment set to 100 % (n = 2 per group, values expressed as mean with standard deviation for error bars; one representative experiment of three independent ones is shown). Due to the semi-quantitative nature of the experiments, no statistical test was performed. C – FRET experiments using COS-1 cells co-transfected with telethonin (aa 1-90) fused to YFP (first row) and titin Z1Zr3 fused to CFP (second row). Images pre- and post-bleach are shown, FRET ratios are shown in the third row. Inserts show magnification of indicated area. Scale bar represents 10 microns. D – Quantification of FRET efficiency for titin Z1Zr3 WT/A178D and telethonin pairs. The A178D mutation reduces the FRET efficiency from approx. 0.15 to 0.01 indicating a dramatic loss of binding ability (WT n=20 and A178D n=26 cells; * $p < 0.0001$ unpaired Student’s t-test).

American Heart Association.
Circulation
Cardiovascular Genetics

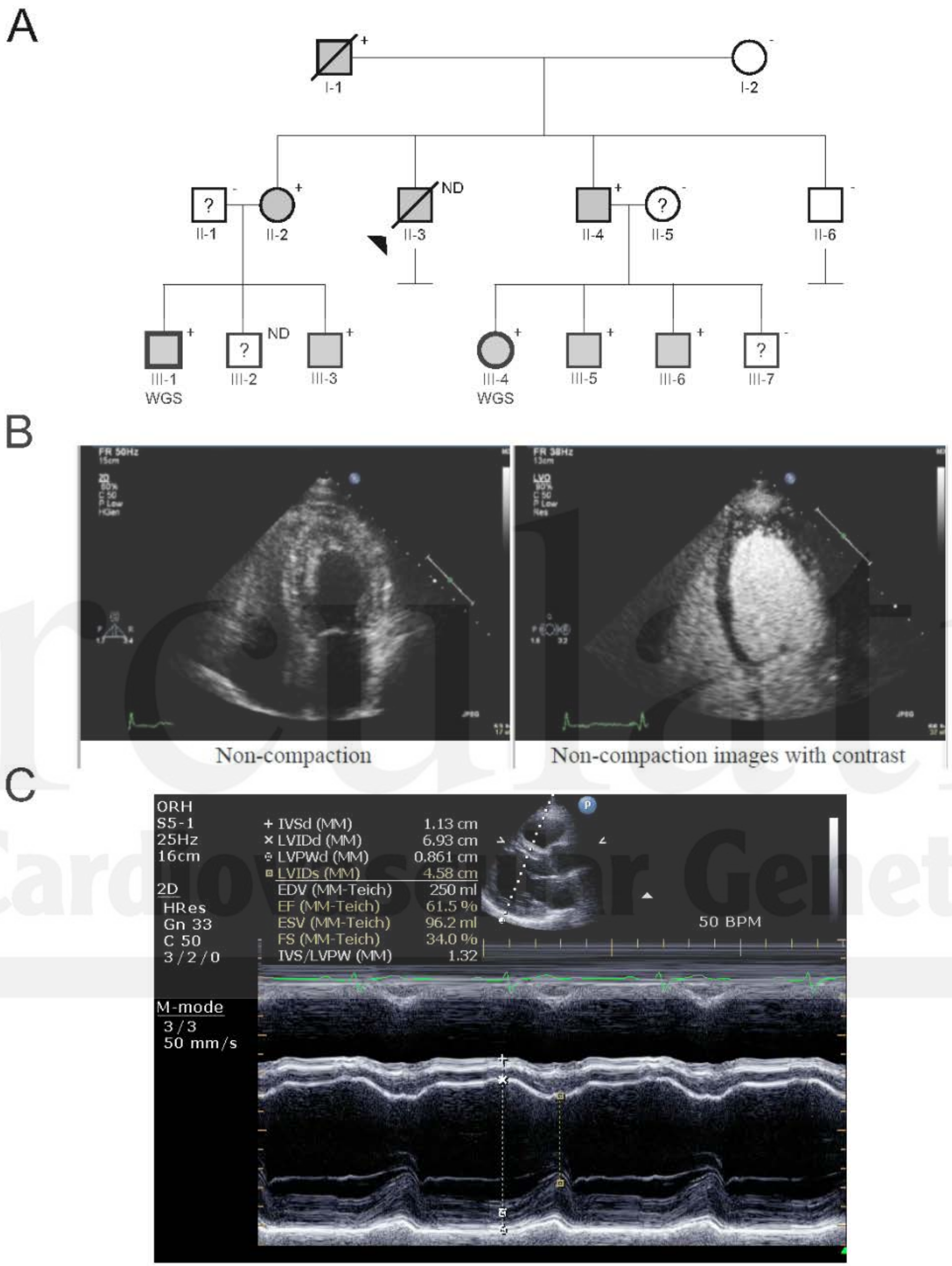

American Heart Association
Circulation
Cardiovascular Genetics

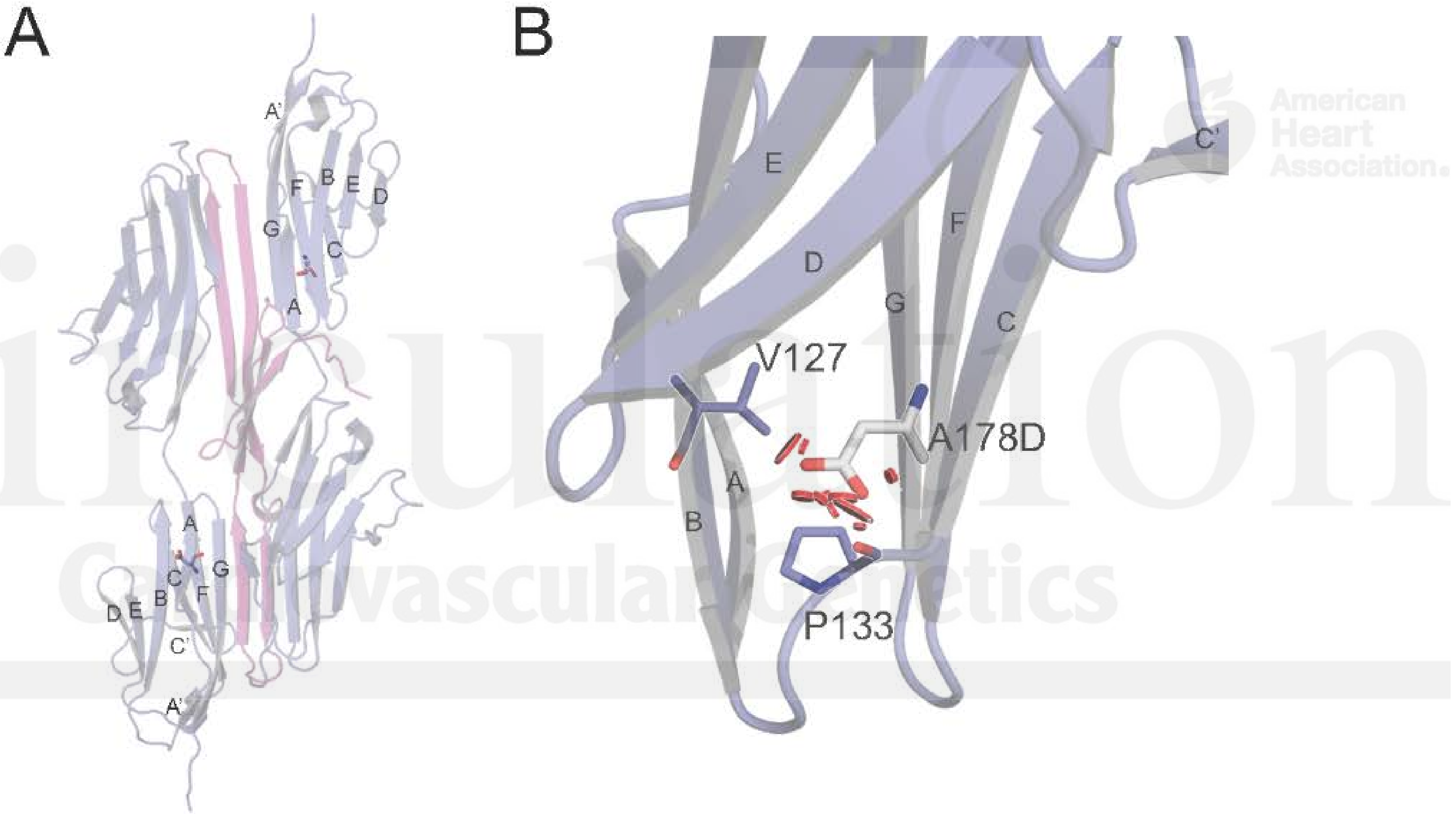

American Heart Association.
Circulation
Cardiovascular Genetics

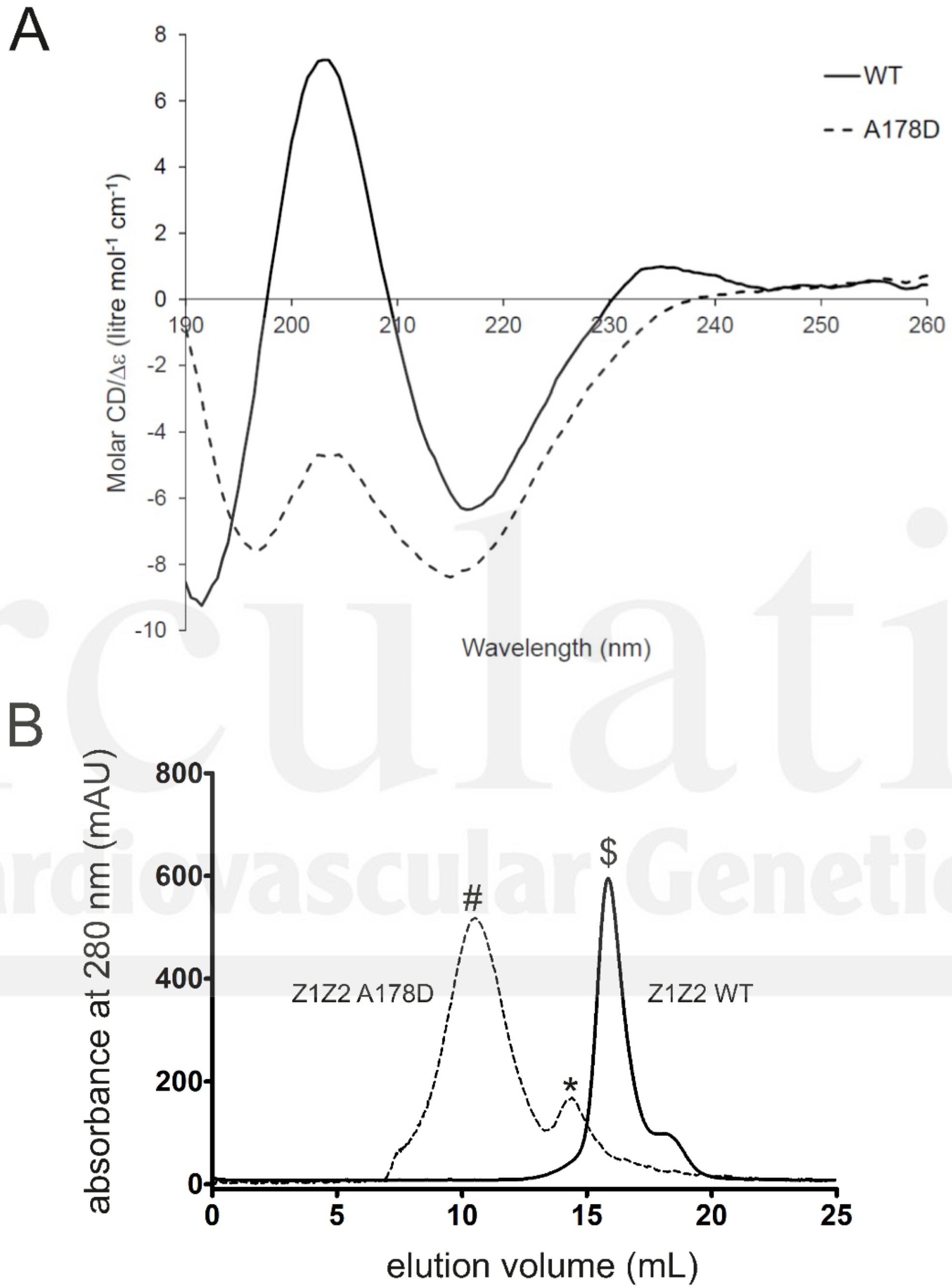

Circulation Cardiovascular Genetics
American Heart Association.

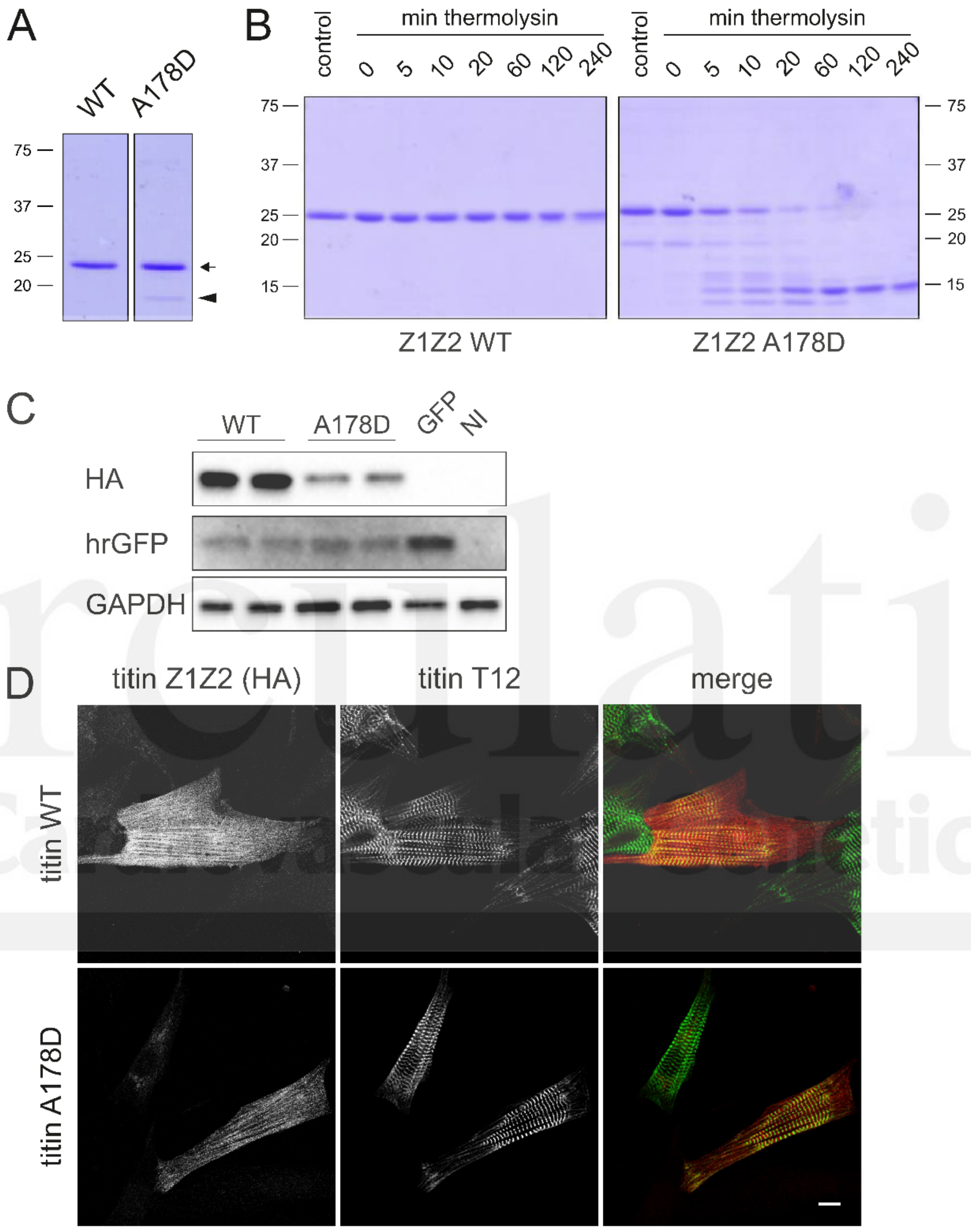

American Heart Association
Circulation Cardiovascular Genetics

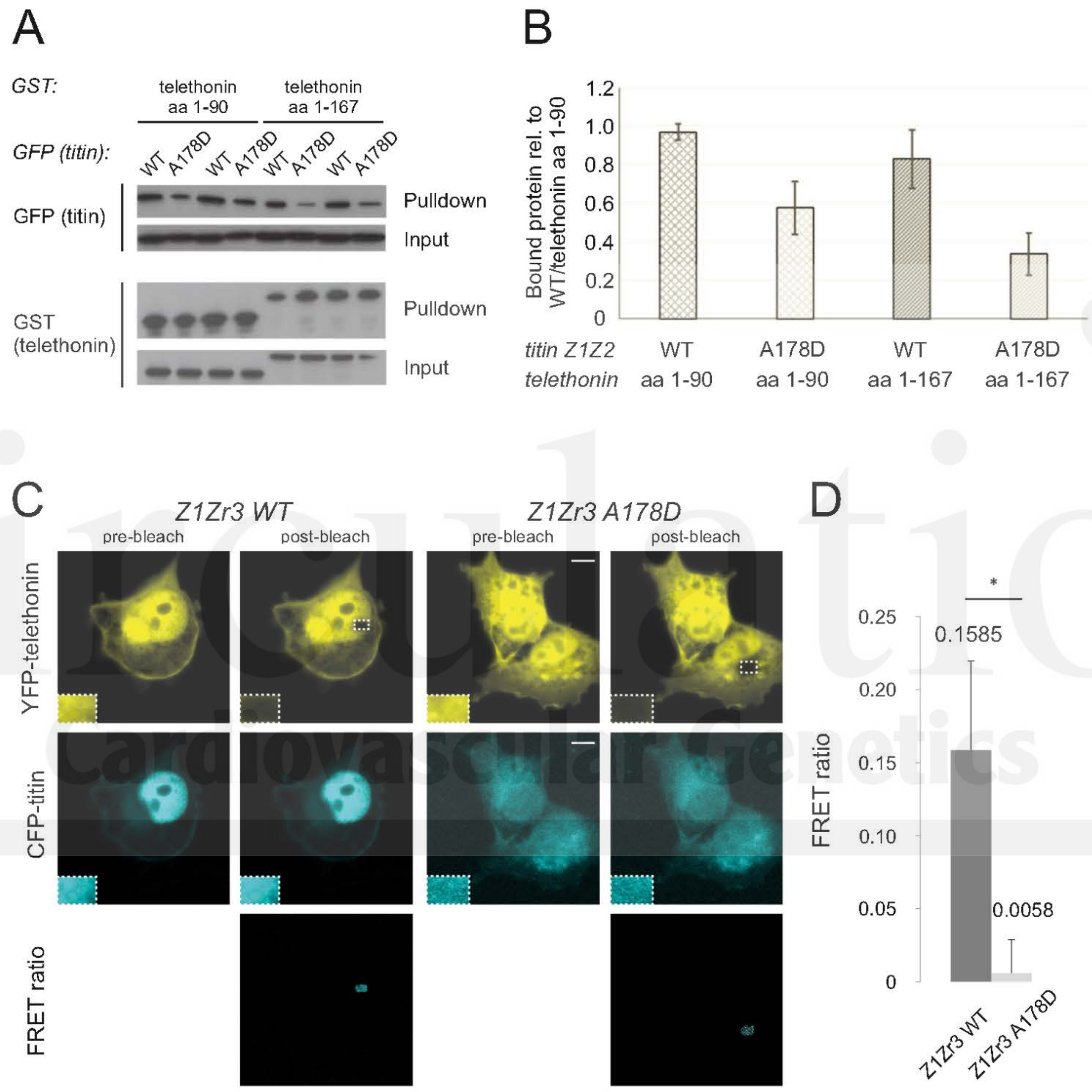

American Heart Association.
Circulation
Cardiovascular Genetics
